# Autonomous Parameter Balance in Population-Based Approaches: A Self-Adaptive Learning-Based Strategy

**DOI:** 10.3390/biomimetics9020082

**Published:** 2024-01-31

**Authors:** Emanuel Vega, José Lemus-Romani, Ricardo Soto, Broderick Crawford, Christoffer Löffler, Javier Peña, El-Gazhali Talbi

**Affiliations:** 1 Escuela de Ingeniería Informática, Pontificia Universidad Católica de Valparaíso, Avenida Brasil 2241, Valparaíso, Valparaíso 2362807, Chile; broderick.crawford@pucv.cl (B.C.); christoffer.loffler@pucv.cl (C.L.); javier.pena.r@mail.pucv.cl (J.P.); 2 Escuela de Construcción Civil, Pontificia Universidad Católica de Chile, Avenida Vicuña Mackenna 4860, Macul, Santiago 7820436, Chile; jose.lemus@uc.cl; 3 CNRS/CRIStAL, University of Lille, 59655 Villeneuve d’Ascq, France; el-ghazali.talbi@univ-lille.fr

**Keywords:** machine learning, hybrid approach, self-adaptive strategies, optimization, 68T05, 68T02, 68R05

## Abstract

Population-based metaheuristics can be seen as a set of agents that smartly explore the space of solutions of a given optimization problem. These agents are commonly governed by movement operators that decide how the exploration is driven. Although metaheuristics have successfully been used for more than 20 years, performing rapid and high-quality parameter control is still a main concern. For instance, deciding the proper population size yielding a good balance between quality of results and computing time is constantly a hard task, even more so in the presence of an unexplored optimization problem. In this paper, we propose a self-adaptive strategy based on the on-line population balance, which aims for improvements in the performance and search process on population-based algorithms. The design behind the proposed approach relies on three different components. Firstly, an optimization-based component which defines all metaheuristic tasks related to carry out the resolution of the optimization problems. Secondly, a learning-based component focused on transforming dynamic data into knowledge in order to influence the search in the solution space. Thirdly, a probabilistic-based selector component is designed to dynamically adjust the population. We illustrate an extensive experimental process on large instance sets from three well-known discrete optimization problems: Manufacturing Cell Design Problem, Set covering Problem, and Multidimensional Knapsack Problem. The proposed approach is able to compete against classic, autonomous, as well as IRace-tuned metaheuristics, yielding interesting results and potential future work regarding dynamically adjusting the number of solutions interacting on different times within the search process.

## 1. Introduction

Metaheuristics (MH) correspond to a heterogeneous family of algorithms, and multiple classifications have been proposed, such as single-solution, population-based, and nature-inspired [1]. In addition, it is well-known that the tuning of their key components, such as movement operators, stochastic elements, and parameters, can be the objective of multiple improvements in order to achieve a better performance. In this regard, dynamically adjusting parameters such as the population size is an important topic to the scientific community, which focuses its research on the employment of population-based approaches in order to solve hard optimization problems. This parameter can be considered as one of the most transversal issues to be defined on population-based algorithms. Nevertheless, it can be illustrated that this issue can be the most difficult parameter to settle on MH [2]. Moreover, the real impact behind the number of agents has been rarely addressed and proved to depend on several scenarios [3]: for instance, variants designed to perform in a particular study case, approaches designed to perform on a specific application, and approaches designed to tackle high-dimensional problems. Thus, in order to improve the arduous task in controlling this parameter, we propose a novel self-adaptive strategy, which aims to dynamically balance the amount of agents by analyzing the dynamic data generated on run-time solving different discrete optimization problems. In the literature, this kind of strategy has developed a solid foot in the optimization field, and in particular on evolutionary algorithms, where the generalized ideas include convergence optimization, global search improvements, and high affinity on parallelism, among others [4,5,6,7]. For instance, it is well-known that the harmony search algorithm has drawbacks such as falling in local optima and premature convergence. However, these issues have been tackled by improvements on its internal components, data management, tuning parameters, and search process [8,9,10]. The real well-known issue which exists to this day concerns the proposition of tailored/fitted solutions focused to perform under certain conditions, constrained to a defined environment, tackling a clear objective, and a specific problem or even specific instances within a problem [3]. Moreover, in the literature there exist a wide number of proposed MH. However, a recurrent scenario is that most advances and improvements proposed in the state-of-the-art are focused on well-known algorithms, such as the works regarding the population size and other parameters in Particle Swarm Optimization (PSO) [11,12,13]. In this context, the proposed approach, named Learning-based Linear Population (LBLP), aims for an improvement in the performance achieved by a pure population-based algorithm thanks to the influence given by the incorporation of a learning-based component balancing the agents on run-time. In addition, the interaction between MH and Machine Learning (ML) has attracted massive attention from the scientific community given the great results yielded on their respective fields [14,15,16,17,18].

The design proposed in this work includes the definition of three components which are mainly based on ideas and techniques from the optimization and ML fields [19]. In this context, the first component focuses on the management of major issues concerning population-based related tasks, such as generation of the initial population, intensification, diversification, and binarization. In this first attempt, we employ the Spotted Hyena Optimizer (SHO) algorithm [20], which has proved to be a good option solving optimization problems [21,22,23,24,25,26]. Regarding the second component, the main objective is the management of the dynamic data generated. This component includes two major tasks, which are the management of the data-structures behind LBLP and the management of the learning-based method. The learning process is carried out by a statistical modeling method ruled by the means of multiple linear regression. In this context, the process in control of the population size will be influenced by the knowledge generated through this process. The third proposed component concerns the management of parameters and agents used by LBLP while carrying out the solving process. In this regard, three major tasks are performed through the search process, the selection mechanism, control of probabilities, and increase/decrease of solutions within the population. The objective behind the selection mechanism includes the proper choice of a population size to perform for a certain amount of iterations on run-time. The design behind the mechanism follows a Monte Carlo simulation strategy. The second task concerns the control of parameters such as the probabilities employed. In addition, the third task carries out the generation-increase and the removal of solutions.

In order to test the performance and prove the viability or our proposed hybrid approach, we solve three well-known optimization problems, named the Manufacturing cell design problem (MCDP) [27], the Set covering problem (SCP) [28], and the Multidimensional knapsack problem (MKP) [29]. The illustrated comparison is carried out in a three-step experimentation phase. Firstly, we carry out a performance comparison against reported results yielded by competitive state-of-the-art algorithms. Secondly, we compare against a pure implementation of SHO assisted by IRace, which is a well-known parameter tuning method [30]. Thirdly, we compare the results obtained by the pure implementation of SHO vs. our proposed hybrid. Finally, we illustrate the interesting experimental results and discussion, where the proposed LBLP achieves good performance, proving to be a good and competitive option to tackle hard optimization problems.

The main contributions and strong points in the proposal can be described as follows.

- Robust self-adaptive hybrid approach capable of tackling hard optimization problems;
- Online-tuning/Control of a key issue in population-based approaches: Adapting population size on run-time;
- The hybrid approach successfully solved multiple hard optimization problems: In the experimentation phase, great results were achieved solving the MCDP, SCP, and MKP by employing an unique set of configuration values;
- Scalability in the first component designed: This work proved great adaptability given to the employed population-based algorithm. This allows the incorporation of several movement operators from different population-based algorithms in order to be instantiated by the approach to perform (parallel approach);
- Scalability in the third designed component: This work demonstrated significant benefits derived from the dynamic data generated through the search. The proposed design allows for the incorporation of different techniques, such as multiple supervised and deep learning methods.

The rest of this paper is organized as follows. The related work is introduced in Section 2. In Section 3 we illustrate the proper background in order to fully understand the proposed work and optimization problems solved. The proposed hybrid approach is explained in Section 4. Section 5 illustrates the experimental results. Finally, we conclude and suggest some lines of future research in Section 6.

## 2. Related Work

The proposed self-adaptive strategy has been designed by the interaction of multiple components from the optimization and machine learning field. In the literature, this kind of proposal has been known as hybrid approaches, which aims to incorporate knowledge from data and experience to the search process while solving a given problem. This line of investigation has received noteworthy attention from the scientific community and multiple taxonomies have been reported [19,31].

Preliminary works concerning machine learning at the service of optimization methods has been a trendy approach in recent years. In Ref. [32], a hybrid approach conformed by TS and Support Vector Machine (SVM) was proposed. The objective was to design an approach capable of tackling hard combinatorial optimization problems, such as Knapsack Problem (KP), Set Covering Problem (SCP), and the Traveling Salesman Problem (TSP). The proposed hybrid defined decision rules from a corpus of solutions generated in a random fashion, which were used to predict high quality solutions for a given instance and lead the search. However, the complexity behind the designed approach is a key factor and authors highlight the arduous and time consuming tasks, such as the knowledge needed to build the corpus, and the extraction of the classification rules. In addition, more recent hybrids that integrate self-adaptive strategies in their process have been receiving significant attention given the achieved results. In Ref. [9], an ensemble learning model focused on detecting fake news was proposed. This hybrid includes off-line process and online process. The authors proposed the incorporation of a self-adaptive harmony search at the off-line process in order to modify the weight of four defined training models based on different CNN versions. However, the issues persist to this end, such as computation complexity, resources, and solutions being tailored for an specific objective.

The objective behind the proposed approach concerns the improvement in the performance of a pure population-based algorithm based on the proper control of parameters [33]. This work gives emphasis on the population size value, which is well-known for being a key parameter defined by all swarm-based approaches. In this regard, similar objectives can be observed in Refs. [22,34], where the authors proposed two hybrids that follow the same objective. The approaches employ Autonomous Search (AS) to assist the MH Human Behavior-Based Optimizer (HBBO) and SHO, respectively. AS is described as a reactive process that lets the solvers automatically reconfigure their parameters in order to improve when poor performances are detected. Nevertheless, data-driven hybrids that follow an equal objective are scarce. Currently, the body research related to this work focuses on classification, clustering, and data mining techniques. In this context, the authors in Ref. [35] proposed a hybrid framework based on the Co-evolutionary Genetic Algorithm (CGA) supported by machine learning. They employed inductive learning methods from a group of elite populations in order to influence the population with lower fitness. Their objective was to achieve a constant evolution of agents with low performance through the search. The learning process was carried out by the C4.5 and CN2 algorithms in order to perform the classification. Regarding data mining-based approaches, in Ref. [36], a hybrid version of ant colony optimization that incorporates a data mining module to guide the search process was proposed. Regarding the usage of clustering-based methods, Streichert et al. [37] proposed the clustering-based nitching method. The main objective behind the proposed approach was to identify multiple global and local optima in a multimodal search space. The model is employed over well-known evolutionary algorithms, and the aim of the model was the preservation of diversity using a multi population approach.

The proposed hybrid brings inspiration from multiple ideas described as follows. Firstly, we propose a hybrid approach that is capable of solving different optimization problems. In addition, the main objective concerns the design of a self-adaptive strategy in order to dynamically adjust and control a key parameter such as the population size on population-based approaches. In this regard, we detected a scarce number of illustrated works that focus their efforts on this issue. In the literature, the objective of most proposed works concern the tuning of parameters. The values are adjusted before the execution of the algorithm, usually through a number of previous runs. However, the proposed work adjusts the parameter values on-the-fly. In this context, a control method chooses a set of values for the optimization algorithm to perform in a given amount of time. The performance achieved is properly measured. Thus, the control method is able to know how good that choice was. These steps are repeated, and the aim is to maximize the chances of success by making the best decisions in the optimization process. On the other hand, although there is a clear presence of well-known learning-based methods such as clustering and classification, regression analysis is hardly employed, leaving out highly potential models that can tackle the presented issue. Lastly, we highlight the promising results obtained by solving three different hard optimization problems, which are illustrated in Section 5. In this regard, most proposed approaches in the literature are problem-oriented. Nevertheless, one of our objectives is for the proposed approach to be nature-friendly for the given problem. Thus, with the presented results a promising contribution to the field is illustrated.

## 3. Background

In this section, we review essential topics needed in order to fully understand the proposed hybrid. Firstly, the main features of population-based methods are presented, followed by the description of the employed SHO algorithm. Secondly, the detailed description of the proposed problems solved in this work are illustrated.

### 3.1. Metaheuristics

The MHs can be described as general-purpose methods that have great capabilities to tackle optimization problems [38]. This heterogeneous family of algorithms has been the focus of several works as a consequence of their attractive features such as the capability to tackle hard optimization problems in a finite computational time, achieving close-to-optimal solutions [39]. In the literature, subgroups from this family have been identified thanks to different criteria given the features of the proposed algorithms. Firstly, single solution algorithms were designed to carry out the transformation of a single solution during the search. Well-known examples are the local search [40], simulated annealing [41], etc. On the other hand, population-based algorithms focus on the transformation of multiple solutions during the search. In this context, all the agents/solutions in the population interact between them and evolve. Well-known algorithms are the shuffle frog leaping algorithm [42], ant colony optimization [43], gray wolf optimizer [44], and so on. Another big family of proposed algorithms consist of nature-inspired approaches. They are born as metaphors that define their behaviors on the basis of nature. For instance, the genetic algorithm [45], memetic algorithm [46], and differential evolution [47]. Additionally, an inverse phenomena can be described for non-natural algorithms, such as imperialist competitive algorithm [48], and several subgroups of algorithms designed from multiple fields, such as music, physics, and so on. However, all the proposed algorithms in this heterogeneous family share between them equal concepts in their design, such as ideas, components, parameters, and so on.

#### 3.1.1. Spotted Hyena Optimizer

In this work, we employ the SHO algorithm, which is a population-based MH that follows clustering ideas in their performance and has proved to be a good option for solving optimization problems. The main concept behind this algorithm is the social relationship between spotted hyenas and their collaborative behavior, which was originally designed to optimize constraint and unconstrained design problems. Regarding the description and equations of the movement operators, at the beginning, encircling prey is applied. The objective is to update the position of each agent towards the current best candidate solution in the population. In order to carry out the perturbation on each agent, we employ Equations (1) and (2). In (1), $D_{h}$ is the distance between the current agent (*P*) and the actual best agent in the population ($P_{p}$). In addition, in Equation (2), we compute the update of the current agent. In both equations, *B* and *E* correspond to co-efficient vectors; they are computed as illustrated in Equations (3) and (4), where $rd_{1}$ and $rd_{2}$ are random [0, 1] vectors.

(1) $$D_{h}=\left|B\cdot P_{p}\left(x\right)-P\left(x\right)\right|$$

(2) $$P\left(x+1\right)=P_{p}\left(x\right)-E\cdot D_{h}$$

(3) $$B=2\cdot rd_{1}$$

(4) $$E=2h\cdot rd_{2}-h$$

(5) $$h=5-(Iteration*\left(5/Max_{iteration}\right))$$

The second movement employed is named hunting. The main objective is to influence the decision regarding the next position of each agent and the main idea is to compose a cluster towards the current best agent. In order to carry out this movement, we employ Equations (6)–(8). In (6) and (7), $D_{h}$ represents the distance, $P_{h}$ represents the actual best agent in the population, and $P_{k}$ the current agent being updated. Equation (7) illustrates the data-structure that contains the population clustered, where *N* indicates the number of agents.

(6) $$D_{h}=\left|B\cdot P_{h}-P_{k}\right|$$

(7) $$P_{k}=P_{h}-E\cdot D_{h}$$

(8) $$C_{h}=P_{k}+P_{k+1}+...+P_{k+N}$$

Attacking the prey is illustrated as the third movement employed. This operator concerns the exploitation of the search space. In (9), each agent belonging to the cluster $D_{h}$, generated in (8), will be updated.

(9) $$P\left(x+1\right)=C_{h}/N$$

The fourth movement concerns the performance of a passive exploration. The proposed SHO performs with *B* and *E* as co-efficient with random values to force the agents to move far away from the actual best agent in the population. This mechanism improves the global search of the approach. Additionally, SHO was initially designed to work on a continuous space. In order to tackle the MCDP, SCP, and MKP, a transformation of domain is needed and this process is illustrated in the next subsection.

#### 3.1.2. Domain Transfer

In the literature, continuous population-based MH have proved to be very effective in tackling several high complex optimization problems [49]. Currently, the increment in complexity of binary modern industrial problems have pushed new challenges to the scientific community, which have ended up proposing continuous methods as potential options to tackle this domain. For instance, Binary Bat Algorithm [50], PSO [51], Binary Salp Swarm Algorithm [52], Binary Dragonfly [53], and Binary Magnetic Optimization Algorithm [54], among others [55,56,57]. In order to carry out the transformation, binarization strategies have been proposed [51]. In this regard, a well-known employed strategy concerns the Two-step binarization scheme, which as the name implies, is composed of a two step process where transformation and binarization is performed. Firstly, transfer functions were introduced to the field in Ref. [58] with the aim to give a probability between 0 and 1 employing low computational resources. Thus, transfer functions, illustrated in Table 1, are applied to the values generated by the movement operator from the continuous MH. This process achieves these values to be in the range between 0 and 1. Secondly, the application of binarization is carried out, which focuses on the value discretization applied to the output values from the first step. This process decides for a binary value (0 or 1) to be selected. In this regard, classic methods have been described as follows:

1. Standard: If the condition is satisfied, standard method returns 1, otherwise returns 0.
  (10) $$X_{i}^{d}\left(t+1\right)=\left\{\begin{array}{cc} 1, & \;\;\;if\;rand\;\leq T(x_{i}^{d}\left(t+1\right))\\ 0, & \;\;\;otherwise \end{array}\right.$$
2. Complement: If the condition is satisfied, standard method returns the complement value.
  (11) $$X_{i}^{d}\left(t+1\right)=\left\{\begin{array}{cc} \bar{x_{i}^{d}{\left(t+1\right)}^{\prime }}, & \;\;\;if\;rand\;\leq T(x_{i}^{d}\left(t+1\right))\\ 0, & \;\;\;otherwise \end{array}\right.$$
3. Static probability: A probability is generated and evaluated with a transfer function.
  (12) $$X_{i}^{d}\left(t+1\right)=\left\{\begin{array}{cc} 0, & \;if\;T(x_{i}^{d}\left(t+1\right))\leq \alpha \\ X_{i}^{d}{\left(t+1\right)}^{\prime }, & \;if\;T\left(x_{i}^{d}\left(t+1\right)\right)\leq \frac{1}{2}\left(1+\alpha \right)\\ 1, & \;if\;T\left(x_{i}^{d}\left(t+1\right)\right)\geq \frac{1}{2}\left(1+\alpha \right) \end{array}\right.$$
4. Elitist Discretization: Method Elitist Roulette, also known as Monte Carlo, consists of selecting randomly among the best individuals of the population, with a probability proportional to its fitness.
  (13) $$X_{i}^{d}\left(t+1\right)=\left\{\begin{array}{cc} x_{i}^{d}{\left(t+1\right)}^{\prime }, & \;\;\;if\;rand\;\leq T(x_{i}^{d}\left(t+1\right))\\ 0, & \;\;\;otherwise \end{array}\right.$$

In this work, the two-step strategy employed consists of the transfer function $V_{4}$ and the elitist discretization.

### 3.2. Optimization Problems

In this subsection we illustrate a detailed explanation of the three optimization problems tackled by our proposed LBLP.

#### 3.2.1. Manufacturing Cell Design Problem

The Manufacturing Cell Design Problem (MCDP) [59] is a classical optimization problem that finds application in lines of manufacture. In this regard, the MCDP consists of organizing a manufacturing plant or facility into a set of cells, each of them made up of different machines meant to process different parts of a product that have similar characteristics. The main objective is to minimize the movement and exchange of material between cells in order to reduce the production costs and increase productivity. The optimization model is stated as follows. Let:

- M—the number of machines;
- P—the number of parts;
- C—the number of cells;
- i—the index of machines (i = 1, …, M);
- j—the index of parts (j = 1, …, P);
- k—the index of cells (k = 1, …, C);
- $A=[a_{ij}]$—the binary machine-part incidence matrix *M* × *P*;
- $M_{max}$—the maximum number of machines per cell. We selected as the objective function to minimize the number of times that a given part must be processed by a machine that does not belong to the cell that the part has been assigned to. Let:

$$y_{ik}=\{\begin{array}{cc} 1 & \mathrm{if}\mathrm{machine}i\in \mathrm{cell}k;\\ 0 & \mathrm{otherwise}; \end{array}$$

$$z_{jk}=\{\begin{array}{cc} 1 & \mathrm{if}\mathrm{part}j\in \mathrm{family}k;\\ 0 & \mathrm{otherwise}; \end{array}$$

  The problem is represented by the following mathematical model:

$$\;\;Minimize\sum_{k=1}^{C}\sum_{i=1}^{M}\sum_{j=1}^{P}a_{i_{j}}z_{j_{k}}\left(1-y_{i_{k}}\right)\;\;\;\;\;\;\;\;$$

Subject to

$$\;\;\sum_{k=1}^{C}y_{ik}=1\;\;\;\;\;\;\;\;\forall i$$

$$\;\;\sum_{k=1}^{C}z_{jk}=1\;\;\;\;\;\;\;\;\forall j$$

$$\;\;\sum_{i=1}^{M}y_{ik}\leq M_{max}\;\;\;\;\;\;\;\;\forall k$$

In this work, we solved a set of 35 instances from different authors. Each instance has its own configuration, the amounts of machines goes from 5 to 40, parts goes from 7 to 100, and so on. For this experiment, the instances tested have been executed 30 times.

#### 3.2.2. Set Covering Problem

The set covering problem (SCP) is one of the well-known Karp’s 21 NP-complete problems, where the goal is to find a subset of columns in a 1-0 matrix so that they cover all the rows of the matrix at a minimum cost. Several applications of the SCP can be seen in the real world, for instance, bus crew scheduling [60], location of emergency facilities [61], and vehicle routing [62]. The formal definition is presented as follows. Let *m* × *n* be a binary matrix A = ($a_{ij}$) and a positive *n*-dimensional vector C = ($c_{j}$), where each element $c_{j}$ of C gives the cost of selecting the column *j* of matrix A. If $a_{ij}$ is equal to 1, then it means that the row *i* is covered by column j, otherwise it is not. The goal of the SCP is to find a minimum cost of columns in A such that each row in A is covered by at least one column. A mathematical definition of the SCP can be expressed as follows:

$$Minimize\;\;\sum_{j=1}^{n}c_{j}x_{j}$$

$$Subject\;\;to\;\;\;\sum_{j=1}^{n}a_{ij}x_{j}\geq 1,\;\;\;i=1,2,...,m$$

$$x_{j}\in \left\{0,1\right\},\;\;\;j=1,2,..,m$$

where $x_{j}$ is 1 if column *j* is in the solution, otherwise it is 0. The constraint ensures that each row *i* is covered by at least one column. In this work, we solved 65 different instances, which have been organized into 11 sets extracted from the Beasley’s ORlibrary. The employed instances were pre-processed in order to reduce the size and complexity. In this context, multiple pre-processing methods have been proposed in the literature for the SCP [63]. In this work, we used two of them, which have proved to be the most effective: Column Domination (CD) and Column Inclusion (CI). Firstly, the definition of CD concerns a set of rows $L_{j}$ being covered by another column $j^{\prime }$ and $c_{j^{\prime }}$<$c_{j}$. We then say that column *j* is dominated by $c_{j^{\prime }}$, and column *j* is removed from the solution. Second, in CI, the process is described as when a row is covered by only one column after the CD is applied. This means that there is no better column to cover those rows, and therefore this column must be included in the optimal solution. For this experiment, the test instances have been executed 30 times.

#### 3.2.3. Multidimensional Knapsack Problem

Multidimensional Knapsack Problem (MKP) is NP-hard and can be considered as the generalized form of the classic Knapsack Problem (KP). The goal of MKP is to search for a subset of given objects that maximize the total profit while satisfying all constraints on resources. In addition, the KP is a widely-used problem with real-world applications in diverse fields including cryptography, allocation problems, scheduling, and production [64,65]. The model can be stated as follows.

$$Maximize\;\;\sum_{j=1}^{n}c_{j}x_{j}$$

$$Subject\;\;to\;\;\;\sum_{j=1}^{n}a_{ij}x_{j}\leq b_{i},\;\;\;i\in M=1,2,...,m$$

$$x_{j}\in \left\{0,1\right\},\;\;\;j\in N=1,2,...,n$$

where *n* is the number of items and *m* is the number of knapsack constraints with capacities $b_{i}$. Each item *j* requires $a_{ij}$ units of resource consumption in the *i*th knapsack and yields $c_{j}$ units of profit upon inclusion. The goal is to find a subset of items that yields maximum profit without exceeding the resource capacities. In this work, we solved 6 different set instances from the Beasley’s ORlibrary. The details concerning the solved benchmark is illustrated in Table 2.

## 4. Proposed Hybrid Approach

In this section we describe the details concerning the proposed hybrid: the main ideas, motivations, and design. Firstly, a general description of the process carried out is presented. In Section 4.2 we describe a more detailed methodology behind LBLP.  Section 4.3, describes the main ideas, objectives, and techniques employed in the design of the proposed components. Finally, Section 4.4 illustrates the proposed algorithms.

### 4.1. General Description

The proposed LBLP follows a population-based solving strategy, which concerns multiple agents evolving in the solution space, intensification and diversification are performed, and the process is terminated when a threshold defined as an amount of iteration is met. Dynamically the adjusting parameters, especially population size, is an important topic that continues to be of growing interest to the natural computation community. In Ref. [3], the authors carried out a complete analysis of different implementations of PSO in order to define the perfect number of agents to perform. However, they highlighted that the same configuration will not necessarily fit each optimization problem or even each instance of the same problem. In this proposal we employ a population-based algorithm and consequently improve the performance by modifying the population size on run-time. This proposed modification is designed by the means of a learning component based on regression, which transforms all the yielded results employing different population sizes during solving time. Thus, the modifications are managed based on the possible best performance that can be achieved by employing a certain size as a population value. In this context, this whole process is governed by two parameters that are used as thresholds in order to carry out different tasks for LBLP: (1) The instance for a new population size to perform and (2) the instance when the knowledge needs to be generated. The first threshold is named α, which decides when the selection process will be carried out. This process will be selecting a suitable population size to perform. The second proposed parameter is named β, which manages when the regression analysis needs to be performed. The steps comprehending the proposed LBLP are described as follows:

**Step** **1:** Set the initial parameters for the population-based algorithm and the regression analysis.
**Step** **2:** Select the initial population size to perform.
**Step** **3:** Generate initial population.
**Step** **4:** while the termination criteria is not met.
**Step** **4.1:** Carry out intensification and diversification on the population.
**Step** **4.2:** Management of dynamic data generated.
**Step** **4.3:** Check if β amount of iteration has been met.
**Step** **4.3.1:** Perform regression analysis.
**Step** **4.3.2:** Management knowledge generated.
**Step** **4.4:** Check if α amount of iteration has been met.
**Step** **4.4.1:** Perform the selection mechanism.
**Step** **4.4.2:** Perform the balance of population.
**Step** **4.5:** Update the population-based algorithm’s parameters.

### 4.2. Methodology

The proposed LBLP defines four different population sizes as schemes to be selected to perform during the search. The initial probability given to each scheme to be selected is equally defined. For instance, if we configure four different size values, their initial probability to be picked corresponds to 0.25. Thus, at iteration 1, the selection mechanism (given by the Roulette selector component) will be choosing a scheme, and this selected value is the one to be performed in the next α iterations. In addition, in each iteration, the component managing the movement operators (given by the Driver component) will be sequentially carrying out diversification and intensification within the agents on the search space. This process generates dynamic data on each iteration that is sorted and stored, and this recollected data will be processed when the threshold β is met, where regression is applied and knowledge is generated. This learning process concerns the results yielded by the regression and the value interpretations, where the scheme with the best computed forecasting fitness value is selected and rewarded as the winner. In this regard, if this probability is selected it will be boosted by the model.

### 4.3. LBLP Components

In this subsection, we present a detailed explanation and definition of each component proposed in our first attempt designing LBLP.

#### 4.3.1. Component 1: The Driver

The solving strategy employed by the proposed hybrid follows a population-based design. This component brings inspiration from the optimization field in order to search in the solution space of a given problem. The objective behind this component includes the generation of initial/new population (solutions), intensification, diversification, and binarization. In this first attempt proposing LBLP, we employ SHO mostly because it can be identified as a modern MH, outside of the well-known PSO, differential evolution, and genetic algorithms. In addition, the selection was based on the expertise of the research team. Nevertheless, in future upgrades, the incorporation of several algorithms smartly-selected to perform on run-time will be considered. Regarding the domain transfer process, the driver will be carrying the two-step strategy over the solutions generated. The strategy performed was function $V_{4}$ and the elitist discretization, which has already been proved to perform.

#### 4.3.2. Component 2: Regression Model

This component is the key factor in LBLP. It concerns the analysis, storage, and decision making over the dynamic data generated. In this regard, while a scheme is performing, the regression model will be storing and indexing their respective fitness values achieved. Concerning the data-structure employed, in this work they were designed as vectors, but a more generalized description is presented as follows.

$$DSfit_{i}^{d}=\left[value_{i}^{d},value_{i+1}^{d},\ldots ,value_{n}^{d}\right]\;\;\mathrm{with}\;\;i=\left\{1,2,\ldots ,n\right\}$$

$$DSprob_{i}^{d}=\left[value_{i}^{d},value_{i+1}^{d},\ldots ,value_{n}^{d}\right]\;\;\mathrm{with}\;\;i=\left\{1,2,\ldots ,n\right\}$$

$$DSsol_{i}^{d}=\left[value_{i}^{d},value_{i+1}^{d},\ldots ,value_{n}^{d}\right]\;\;\mathrm{with}\;\;i=\left\{1,2,\ldots ,n\right\}$$

$$DSrank_{i}^{d}=\left[value_{i}^{d},value_{i+1}^{d},\ldots ,value_{n}^{d}\right]\;\;\mathrm{with}\;\;i=\left\{1,2,\ldots ,n\right\}$$

where $DSfit_{i}^{d}$ stores the fitness values reached by the agents of each scheme performed. $DSprob_{i}^{d}$ concerns the data-structure with the probabilities for each scheme to be selected. $DSsol_{i}^{d}$ represents the data-structure which stores the corresponding solutions for each regression analysis carried out. The data-structure $DSrank_{i}^{d}$ concerns the ranking for each scheme regarding the best values reached. In addition, *d* represents the number of schemes designed to be employed by LBLP. Regarding the regression analysis, it is carried out by the means of linear regression, where the fitted function is of the form:

(14) y=wx+b

where *y* corresponds to the dependent variable, which is the fitness and value to predict. *x* represents the independent variable, which corresponds to the scheme performed. In this simple linear regression model, we present the close relationship between the performance and population size, which is employed through search. Regarding our proposed learning-model, we define four fitted functions for each scheme defined in this work, and they are represented as follows.

$$y_{PI-Grade1}=w_{i}x_{PI-Grade1}+b_{i}$$

$$y_{PI-Grade2}=w_{i}x_{PI-Grade2}+b_{i}$$

$$y_{PI-Grade3}=w_{i}x_{PI-Grade3}+b_{i}$$

$$y_{PI-Grade4}=w_{i}x_{PI-Grade4}+b_{i}$$

In order to solve these functions, we employ the least squares method which is a well-known approach used in the regression field. The outputs of the mentioned analysis goes to $DSsol_{i}^{d}$, where in order to select the winner scheme the model takes the following decision:

(15) $$f\left(DSprob_{i}^{d}\right)=MIN\left(DSsol_{i}^{d}\right)$$

where the probabilities concerning each scheme, stored in $DSprob_{i}^{d}$, will be updated taking in consideration Equation (15) and $DSrank_{i}^{d}$. Thus, this process will be addressed by the selection of the best prognostic regarding fitness defined by the four linear models, and the best result will be given “priority”. A practical example can be described as follows: At the beginning, in each iteration, the approach will select a scheme using a probabilistic roulette. For a four-way scheme, the initial probabilities for each scheme to be selected was in a 25%–25%–25%–25% ratio. Additionally, the regression model is always storing and sorting the fitness values and agents on run-time. When the threshold is met, Equation (15) analyses the prognostic achieved and gives the winning scheme a higher probability to be chosen. For instance, we designed a ratio of 55%–15%–15%–15%.

#### 4.3.3. Component 3: Roulette Selector

The idea associated behind this component corresponds to a roulette system, where the main objective concerns the probabilistic selection mechanism behind the agents performing on run-time. In this work, a 4-way scheme defining 4 different population sizes (20, 30, 40, and 50 agents) is employed. In the literature, the perfect number of agents to be employed has been an everlasting discussion within the scientific community. In this context, in Ref. [3], the reasoning for the selection goes after the complexity such as the high-dimensional or unimodal problems, a designed topology of the proposed approach, and for approaches tackling very large tasks. Thus, the definition of this parameter value concerns an adjusting-testing process. In this work we follow the first standard recommendation given by the authors, which is between 20 and 50 agents. In future upgrades to be proposed for LBLP, new configuration will be employed.

Regarding the selection mechanism, the schemes are placed and selected by their assigned probabilities. The initial probability of each scheme to be selected is defined as follows.

$$p_{i}=\frac{1}{N}\;\;\;\;\mathrm{and}\;\;i=\left\{1,2,\ldots ,N\right\}$$

where *N* corresponds to the number of schemes designed for the approach. Thus, in a 4-way scheme they are described as follows.

$$\frac{1}{N_{PI-Grade1}}+\frac{1}{N_{PI-Grade2}}+\frac{1}{N_{PI-Grade3}}+\frac{1}{N_{PI-Grade4}}=1$$

The probabilities for each scheme will be modified by the regression model after the corresponding analysis on the dynamic data generated is carried out on run-time.

### 4.4. Proposed Algorithm

In this section, we illustrate a detailed description of the proposed Algorithm 1.
**Algorithm 1** *Proposed LBLP*1:*The driver: set initial parameters required for movement operators*2:*Regression Model: set internal parameters, DSfit, DSprob, DSrank, DSsol*3:*The driver: generate initial population*4:*Roulette selector: select initial scheme to perform*5:**while**(i≤MaximumIteration)**do**6:    *The driver: perform intensification family of operators*7:    *The driver: perform diversification family of operators*8:    *Regression Model: store and check values for DSfit*9:    **if** best value was reached performing scheme selected by Roulette selector **then**10:        *Regression Model: check and update values for DSrank*11:    **end if**12:    **if** threshold β is met **then**13:        *Regression Model: perform regression analysis*14:        *Regression Model: update DSsol*15:        *Regression Model: check MIN(DSsol)*16:        *Regression Model: update DSprob*17:    **end if**18:    **if** threshold α is met **then**19:        *Roulette selector: select scheme to perform*20:        **if** check number of agents by the scheme selected **then**21:           *Roulette selector: balance the population*22:        **end if**23:    **end if**24:**end while**

## 5. Experimental Results

In this section, we describe the experimentation process carried out to evaluate the performance of our proposed LBLP. In this context, a two-step experimentation phase was designed in order to test the competitiveness. Firstly, we compare against state-of-the-art algorithms solving the MCDP, SCP, and MKP. In the second step, we compare the results obtained by our LBLP against implementations based in SHO + IRace, and classic SHO. Additionally, the results are evaluated using the relative percentage deviation (RPD). The RPD quantifies the deviation of the best value obtained by the approach from $S_{opt}$ for each instance. The configuration employed is illustrated in Table 3, and we highlight the good performance achieved.

(16) $$RPD=\frac{\left(S-S_{opt}\right)}{S_{opt}}\times 100$$

### 5.1. First Experimentation Phase

As mentioned before, this subsection illustrates a detailed comparison and discussion of the performance achieved by LBLP against reported data from state-of-the-art algorithms for each problem.

#### 5.1.1. Manufacturing Cell Design Problem

In this comparison, we employ the reported results illustrated by Binary Cat Swarm Optimization (BCSO) [66], Egyptian Vulture Optimization Algorithm (EVOA), and the Modified Binary Firefly Algorithm (MBFA) [67]. In order to have a deeper sample of algorithms related to the proposed work, we also include a Human Behavior-Based Algorithm supported by Autonomous Search approach [34], which focuses on the control of the population size on run-time. In addition, to compare and understand the results, we highlighted in bold the best result for each instance when the optimum is met.

Table 4 illustrates the comparison of the reported results, and the description is as follows. The first column ID represents the identifier assigned to each instance. The $S_{opt}$ depicts the global optimum or best value known for the given instance. Column Best, Mean, and RPD are the given values for best value reached, the mean value of 30 executions, and the relative percentage deviation correspondingly for each approach. Regarding the performance comparison, the lead is clearly dominated by BCSO and the proposed LBLP. Analyzing the values reported in column Best, BCSO gets all 35 best values known, in comparison to 25 values for LBLP. In addition, concerning the median values for column Best in all the instances, LBLP can be placed in second place with 35.14 and the algorithm with the best performance reported is BCSO, which has a median value of 34.51. In this regard, far behind follows MBFA, EVOA, and HBBO-AS which computed 42.54, 50.83, and 55.03 respectively. On the other hand, concerning the median value for column Mean, the proposed LBLP gets first place with 36.00 against a 36.61 reached by BCSO. This can be interpreted as being more robust and consistent in the reported performance. Moreover, we highlight that in several results, the proposed LBLP remains close to the best values known for those instances, which gives room for future improvements. Thus, the overall observations can be described as follows. LBLP does not fall behind against state-of-the-art algorithms specially designed to tackle the MCDP. In addition, the proposed approach achieved better results than HBBO-AS, which can be interpreted as how a population-based approach makes great profit due to the adaptability given by statistical modeling methods.

#### 5.1.2. Set Covering Problem

In this comparison, we made use of the reported results illustrated by binary cat swarm optimization (BCSO) [68], binary firefly optimization (BFO) [69], binary shuffled frog leaping algorithm (BSFLA) [70], binary artificial bee colony (BABC) [71], and binary electromagnetism-like algorithm (BELA) [72]. In addition, we highlighted in bold the best result for each instance when the optimum is met.

Table 5 illustrates the comparison of results achieved by LBLP against the state-of-the-art algorithms specially designed to tackle the SCP; the description is as follows. The column ID represents the identifier assigned to each instance. The $S_{opt}$ depicts the global optimum or best value known for the given instance. Column Best, Mean, and RPD are the given values for the best value reached, the mean value of 30 executions, and the relative percentage deviation correspondingly for each approach. Regarding the performance comparison, between the six approaches the lead is carried by LBLP. In this regard, a closer observation to the median values can be interpreted as follows. The proposed approach achieved the smallest value for column Best and Mean with 197.31 and 199.75, correspondingly. Moreover, the overall performance in the hardest sets of instances, such as groups F, G, and H, is pretty good as we analyzed the RPD values in comparison to BCSO and BELA. We highlight that in several results the proposed LBLP remains close to the best values known for those instances, encouraging us to continue working and further improve our method.

#### 5.1.3. Multidimensional Knapsack Problem

Regarding the MKP, the state-of-the-art algorithms employed include the filter-and-fan heuristic (F& F) [73], Binary version of the PSO algorithm (3R-BPSO) [74], and a hybrid quantum particle swarm optimization (QPSO) algorithm [75]. These approaches were defined in the literature as specifically designed methods to effectively tackle the MKP, and a certain degree of adaptability was designed into their search process on run-time. For instance, the 3R-BPSO algorithm employs three repair operators in order to fix infeasible solutions generated on run-time. In addition, if the results of an algorithm for a set of benchmark instances are not available, the algorithm will be ignored in the comparative study, for instance, 3R-BPSO in mknapcb2 and mknapcb5. In order to compare and understand the results, we highlighted in bold the best result for each instance when the optimum is met.

Table 6 illustrates the reported performance by the state-of-the-art approaches vs. LBLP. The column ID represents the identifier assigned to each instance. The $S_{opt}$ depicts the global optimum or best value known for the given instance. Column Best, Mean, and RPD are the given values for the best value reached, the mean value of 30 executions, and the relative percentage deviation correspondingly for each approach. Regarding the performance comparison, QPSO and LBLP lead the ranking by the reported performance. The QPSO approach reported a total of 21 best known values and LBLP reached 20 optimum values out of 30. However, observing the median values for column best, the proposed LBLP falls behind even against F & F with a 67,179.10 vs. 67,438.93. In this context, this issue can be clearly observed by the RPD values, instances mknapcb2 and mknapcb5 computed 1.47% and 3.13%, respectively, for tests 5.250.04 and 10.250.04. Thus, there exists a considerable distance between the performance for those instances where the best values known is not reached by LBLP. Nevertheless, in this first attempt, LBLP proved to be a competitive approach capable of tackling multiple optimization problems. In addition, this issue encouraged us to further improve and take profit from multiple mechanisms and heuristics to be employed in the design.

#### 5.1.4. Overall Performance in This Phase

**Figure 1:**
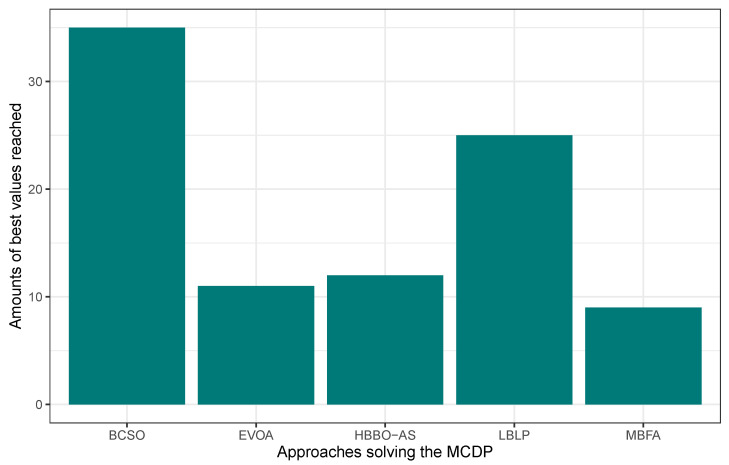
Performance comparison between state-of-the-art approaches vs. LBLP tackling the MCDP.

**Figure 2:**
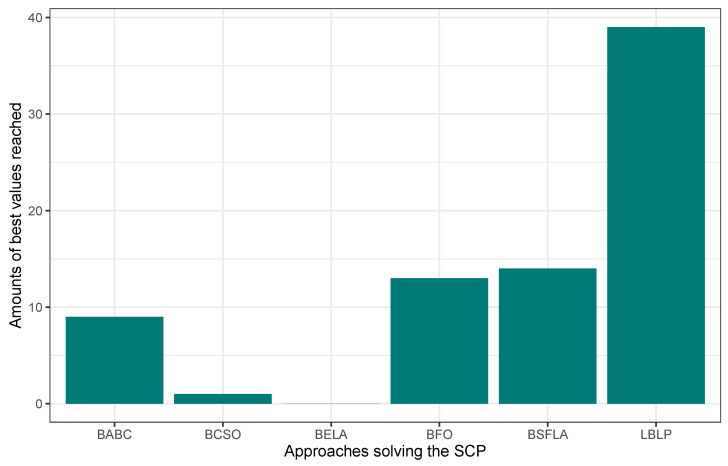
Performance comparison between state-of-the-art approaches vs. LBLP tackling the SCP.

**Figure 3:**
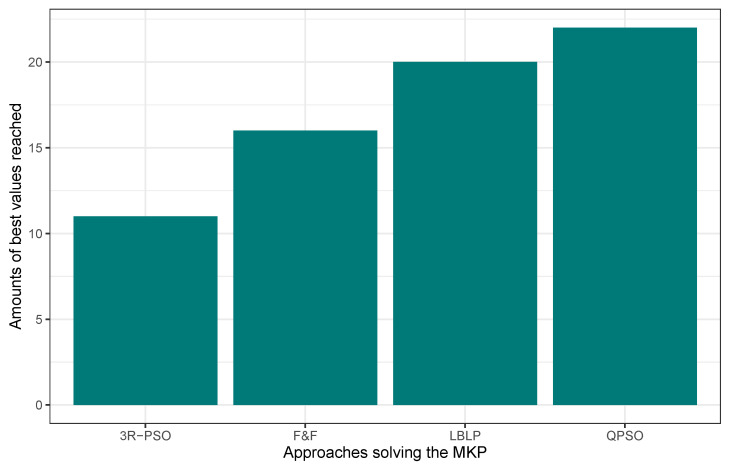
Performance comparison between state-of-the-art approaches vs. LBLP tackling the MKP.

In this first experimentation phase, LBLP was compared against state-of-the-art approaches specially designed to tackle the MCDP, SCP, and MKP. In this context, the proposed hybrid demonstrated a competitive performance for the three different problems tested. Regarding the MCDP, LBLP achieved 25 best values known out of a total of 35, achieving second place overall; see Figure 1. Regarding the SCP, LBLP achieved 39 best values known out of 65, achieving first place; see Figure 2. In addition, it is well-known that optimization methods such as MH are designed to perform in certain environments. Thus, there exists a certain degree of uncertainty when employing such methods to tackle different types of optimization problems. For instance, we can observe the polarized performance reported by BCSO solving the MCDP and SCP. In this regard, this is one of the strong points of our proposition, as the optimization problem to be tackled is not an issue given the adaptability of our proposed LBLP. Regarding the MKP, the proposed LBLP reached second place with 20 best values known out of 30; see Figure 3. Regarding the overall performance, LBLP proved to be competitive. However, we observed an inconsistent performance solving the MKP when the optimum value was not reached. This issue can be interpreted as a consequence of LBLP not taking enough profit from the population size vs. the diversification/intensification relationship and the frequency on which knowledge is opportunely generated. In this first attempt designing LBLP, the approach works with static values for α and β. In this context, a first improvement can be described as the incorporation of a new learning-based component managing the values for α and β on demand. The objective will be to achieve higher adaptability, giving the decision to auto-assign thresholds to perform the scheme-selection mechanism and the regression analysis.

### 5.2. Second Experimentation Phase

In this subsection, we take a closer look at the performance achieved by classic and hybrid approaches. We compare and discuss implementations based on the classic SHO, classic SHO assisted by IRace, and the proposed LBLP. In addition, in order to further demonstrate the improvement given by hybrids in optimization tools, the Wilcoxon’s signed rank (Mann and Whitney 1947) test is carried out. We highlight the improvements, shortcomings, complexity, and robustness observed through the comparison.

#### 5.2.1. Manufacturing Cell Design Problem

In this experimentation, Table 7 illustrates the comparison of results obtained by Classic SHO, Classic SHO assisted by IRace, and LBLP. In addition, in Table 8, a comparison against a hybrid approach is presented. This hybrid was proposed by Soto et al. in Ref. [34] and includes an approach based on the interaction between the population-based human behavior-based algorithm supported by autonomous search algorithm and autonomous search (HBBO-AS), which focuses on the modification of the population. The table description is as follows: column ID represents the identifier for each instance; the $S_{opt}$ depicts the global optimum or best value known for the given instance; column Best, Worst, Mean, and RPD are the given values for best value reached, the worst value reached, the mean value of 30 executions, and the relative percentage deviation correspondingly for each approach. In order to compare and understand the results, we highlighted in bold the best result for each instance when the optimum is met.

Regarding the overall performance of approaches related to SHO, LBLP takes the lead and Classic SHO goes in last place. If we observe the median values, LBLP obtained 35.14 in comparison to 36.09 achieved by Classic SHO, and 35.43 by Classic SHO + IRace for column best. However, Classic SHO + Irace seems to be more consistent as we observed columns Worst and Mean, where 36.37 and 35.90 were the median values achieved against 37.54 and 36.00 reached by LBLP. Nevertheless, the achieved performance can be expressed as hybrid approaches being more competitive than their respective classic algorithms. LBLP demonstrated to be a good option tackling the MCDP and rooms for improvements were observed. In order to further demonstrate the performance by hybrids solving the MCDP, a statistical analysis is carried out. Table 9 illustrates a matrix that comprehends the resulting *p*-values after applying the well-known Average Wilcoxon–Mann–Whitney test for all the instances corresponding to the MCDP. Thus, a *p*-value less than 0.05 means that the difference is statistically significant, so the comparison of their averages is valid, such as LBLP vs. SHO. Concerning the comparison between LBLP and HBBO-AS, the approach led by autonomous search falls clearly behind on all the columns presented. However, new ideas and future interaction between optimization tools are highlighted. For instance, regarding performance metrics, the main job carried out by AS was to detect low performance or repetitive values/patterns on the solution. In this context, new components based on deep learning would clearly be effective at tackling this task.

#### 5.2.2. Set Covering Problem

In this subsection, the results obtained by the three implementations are illustrated in Table 10. The description of the table is as follows: column ID represents the identifier assigned to each instance; $S_{opt}$ depicts the global optimum or best value known for the given instance; column Best, Mean, and RPD are the given values for best value reached, the mean value of 30 executions, and the relative percentage deviation correspondingly for each approach. In order to compare and understand the results, we highlighted in bold the best result for each instance when the optimum is met.

Regarding the best values achieved, LBLP leads with 39 followed by 23 achieved by Classic SHO + IRace, and Classic SHO with 18. This is confirmed by the median values in column best and RPD, where LBLP achieved 197.31 and 1.09, Classic SHO computed 199.89 and 2.24, and Classic SHO + IRace obtained 197.95 and 1.42. Moreover, we highlight that even in the instances where the best values are not met, LBLP stays close to the reported values and this can be corroborated by the small RPD values computed. On the other hand, two interesting phenomenons can be observed in this test. Firstly, the hybridized implementation outperforms the classic approach. In addition, the LBLP median values for column mean can be interpreted as a degree of deficit in robustness. In order to tackle this issue, new improvements will be performed over the regression model and configuration parameters. Nevertheless, LBLP reached most values known and proved to be a competitive option tackling the SCP, which has multiple opportunities to evolve and improve in future works. In order to further demonstrate the performance by hybrids solving the SCP, a statistical analysis is carried out. Table 11 illustrates a matrix that comprehends the resulting *p*-values after applying the well-known Average Wilcoxon–Mann–Whitney test for all the instances corresponding to the SCP. Thus, a *p*-value less than 0.05 means that the difference is statistically significant, so the comparison of their averages is valid, such as LBLP vs. SHO.

#### 5.2.3. Multidimensional Knapsack Problem

In this subsection, the results obtained tackling the MKP are compared and discussed. In Table 12, we illustrate the comparison of results obtained by the three implementation works. In addition, Table 13 illustrates a comparison between the proposed LBLP and LMBP, which is a hybrid architecture based on population algorithm assisted by multiple regression models [76]. The table description is as follows: column ID represents the identifier assigned to each instance; $S_{opt}$ depicts the global optimum or best value known for the given instance; Column Best, Worst, Mean, and RPD are the given values for the best value reached, the worst value reached, the mean value of 30 executions, and the relative percentage deviation correspondingly for each approach. In order to compare and understand the results, we highlighted in bold the best result for each instance when the optimum is met.

Regarding the best values concerning Table 12, the implementation employing IRace leads the overall performance with median values for column Best of 67,268, followed by LBLP with 67,179, and 66,730 for classic SHO. In addition, this is corroborated by the median values computed for column RPD, and IRace achieved 0.27 against a 0.35 for the proposed hybrid. However, the phenomenon observed in this test differs completely in comparison to the ones reported in the previous subsections. The median values reported for column Mean illustrates good robustness in the overall performance of LBLP. On the other hand, the bad results illustrated by Classic SHO + IRace can be interpreted as an inconsistency in the performance and as being trapped in local optima in multiple MKP instances. In order to further demonstrate the performance by hybrids solving the MKP, a statistical analysis is carried out. Table 14 illustrates a matrix that comprehends the resulting *p*-values after applying the well-known Average Wilcoxon–Mann–Whitney test for all the instances corresponding to the MKP. Thus, a *p*-value less than 0.05 means that the difference is statistically significant, so the comparison of their averages is valid, such as LBLP vs. SHO. Regarding results in Table 13, an equal competition is observed, and LMPB is capable of achieving better values for solving instances where LBLP falls behind. Nevertheless, it is interesting to consider designing a more complete or complex learning-based component. We observed that there is no certainty in achieving a good performance with a sole technique solving all the instances. Thus, a proper answer to this issue could be presented by the design of different learning techniques.

#### 5.2.4. Overall Performance in This Phase

**Figure 4:**
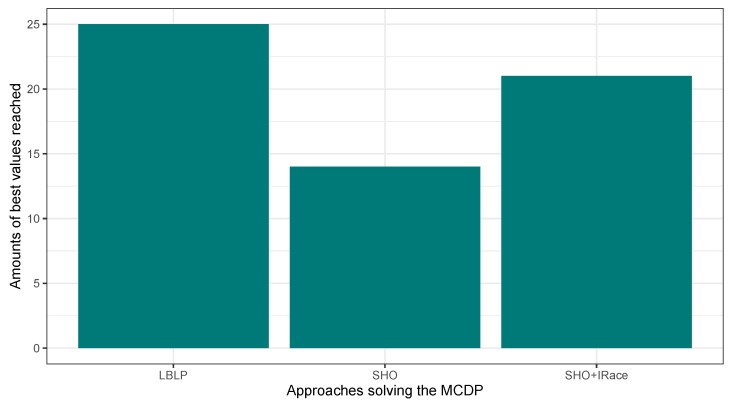
Performance comparison between SHO, SHO assisted by IRace, and LBLP tackling the MCDP.

**Figure 5:**
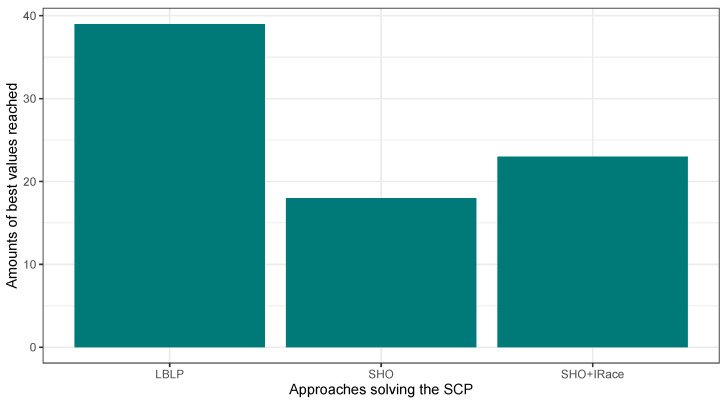
Performance comparison between SHO, SHO assisted by IRace, and LBLP tackling the SCP.

**Figure 6:**
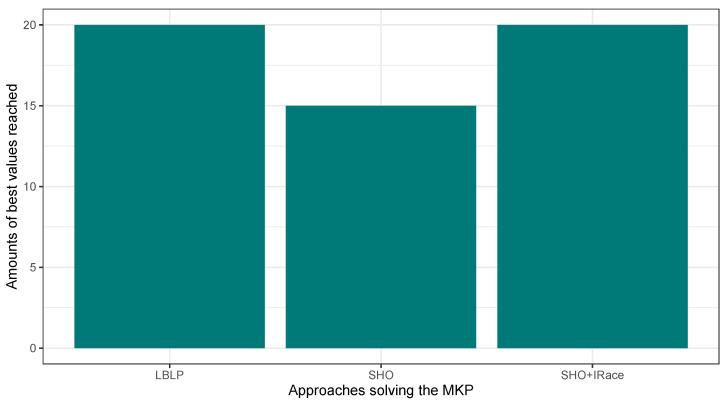
Performance comparison between SHO, SHO assisted by IRace, and LBLP tackling the MKP.

In this second experimentation phase, the proposed LBLP was compared against the Classic SHO and Classic SHO + IRace solving the MCDP, SCP, and MKP. The objective was to verify the improvements blending a learning-based method in the search process of a population-based strategy. In this regard, the proposed LBLP achieved good results solving the optimization problems. Figure 4, Figure 5 and Figure 6 illustrate a performance overview, which ended up corroborating the idea of profiting over dynamic data generated. On the other hand, the good performance demonstrated by Classic SHO + IRace is to be expected. IRace is an off-line method that specializes in the tuning of parameters. Regarding the complexity, users need a certain degree of expertise in R, as the scripts configuration process can be an arduous task, and the implemented optimization tool can be enhanced by IRace. Regarding the proposed approach, LBLP requires the configuration of a scheme, α, and β. In addition, the implementation comprehends a population-based algorithm and well-known statistical modeling methods.

Regarding the observed phenomenons, while solving the MCDP and SCP, LBLP achieved a considerable amount of best values for the column Best but falls behind for the column Mean. Nonetheless, this situation completely changed while solving the MKP. The interpretation can be described in two ways: LBLP asking for a faster response in the learning process and a more detailed configuration of the parameters. Firstly, the parameters proposed in this work are static through the search. This issue was already addressed in Section 5.1.4. Nevertheless, multiple and unexpected events may present themselves while the search is being carried out. Thus, in order for LBLP to answer properly, the first improvement needs to be done over α and β, which controls the scheme selection and the generation of knowledge. On the other hand, concerning the proposed scheme, 4 different values were employed that completely differ from the static values employed by Classic SHO + IRace, such as 41 and 33. In this regard, multiple options as schemes will be added and tested. For instance, 20 different schemes from 20 to 40 agents. Lastly, a more complex scenario can be designed for a further detailed mechanism to be employed by the learning model. The objective is for each defined scheme to implement different α and β values in order to increment the adaptiveness of LBLP.

## 6. Conclusions

In this work, a hybrid self-adaptive approach has been proposed in order to solve hard discrete optimization problems. The main objective was to improve the performance by transforming a general component that exists on all population-based algorithms—the population size. The proposed strategy focuses on the dynamic update of this parameter in order to give high adaptive capacities to the agents, which is governed by a learning-based component that takes profits from their dynamic data generated on run-time. Interesting facts concerning the design are described as follows. As the complexity of the learning component is not high as the statistical modeling methods employed are well-known, the main issue is the novelty in the designed mechanism taking profit of the technique. In this context, movement operators from SHO and linear regression are classic means in their respective fields to solve multiple problems. On the other hand, general complications and drawbacks can be described as follows: computational time incremented, increment of complexity based on the scalability in the designed architecture, and increment at the complexity based on the wide spectrum of optimization problems to be tackled.

Regarding the experimentation carried out, LBLP proved to be a good option in comparison to state-of-the-art methods. We solved three well-known different hard optimization problems: the MCDP, SCP, and MKP, employing a unique configuration set of parameter values for the LBLP. In this context, the first phase helped us to measure at which point LBLP was a viable optimization tool in comparison to already reported approaches. The second phase was meant to highlight the improvements achieved regarding the performance between a pure population-based algorithm vs. the incorporation of a low-level learning-based component of the design. In addition, the competitiveness against reported successful hybrids and parameter-tuned versions of the employed algorithms was highlighted. This is an interesting observation, mainly because of the limitations behind the proposed approach, which concerns the algorithms selected. For instance, if we observe the linear regression, the main drawback is the inclusion of a unique performance metric as an independent variable in the model. In this regard, there are several metrics that exist in the literature and can have different weights during the search, such as bad solutions, percentage of unfeasible solutions, diversity, and amount of feasible solutions generated, among others. Nevertheless, the overall good performance and the given rooms for improvement brings motivation to further exploit this research field. In addition, this work contributed with scientific evidence of hybridized strategies outperforming their classic algorithm, proving to be profitable approaches solving hard optimization problems.

Regarding the phenomenon described in the experimentation phases, future considerations and improvements were discussed. In this regard, two improvements are under consideration: (1) dynamically adjusting values for α and β and (2) multiple and larger ranges for population size values. On the other hand, the well-known drawback generally associated with on-line data-driven methods are the amount, profit, and quality of the data given to the model to properly and timely learn on run-time. Thus, as there is no guarantee for the performance achieved by different learning techniques, it is a major issue to carry out an extensive experimental process employing state-of-the-art regression-based methods. However, this consideration can end up on a considerable increment on solving time in comparison to the ones reported in this work. Thus, the incorporation of an optimizer regarding the computational resources employed on run-time will be a key factor for future proposals.

## Figures and Tables

**Table 1:** Transfer function.

S-Shape	v-Shape
$S_{1}:T\left(x_{i}^{d}\left(t+1\right)\right)=\frac{1}{1+e^{-2x_{i}^{d}\left(t+1\right)}}$	$V_{1}:T\left(x_{i}^{d}\left(t+1\right)\right)=\left|erf\left(\frac{\sqrt{\pi }}{2}x_{i}^{d}\left(t+1\right)\right)\right|$
$S_{2}:T\left(x_{i}^{d}\left(t+1\right)\right)=\frac{1}{1+e^{-x_{i}^{d}\left(t+1\right)}}$	$V_{2}:T\left(x_{i}^{d}\left(t+1\right)\right)=\left|tanh\left(x_{i}^{d}\left(t+1\right)\right)\right|$
$S_{3}:T\left(x_{i}^{d}\left(t+1\right)\right)=\frac{1}{1+e^{\frac{-x_{i}^{d}\left(t+1\right)}{2}}}$	$V_{3}:T\left(x_{i}^{d}\left(t+1\right)\right)=\left|\frac{x_{i}^{d}\left(t+1\right)}{\sqrt{1+{\left(x_{i}^{d}\left(t+1\right)\right)}^{2}}}\right|$
$S_{4}:T\left(x_{i}^{d}\left(t+1\right)\right)=\frac{1}{1+e^{\frac{-x_{i}^{d}\left(t+1\right)}{3}}}$	$V_{4}:T\left(x_{i}^{d}\left(t+1\right)\right)=\left|\frac{2}{\pi }arctan\left(\frac{\pi }{2}x_{i}^{d}\left(t+1\right)\right)\right|$

**Table 2:** Configuration details from MKP instances employed in this work.

ID	Test Problem	Optimal Solution	n	m
mknapcb1	5.100.00	24,381	100	5
	5.100.01	24,274	100	5
	5.100.02	23,551	100	5
	5.100.03	23,534	100	5
	5.100.04	23,991	100	5
mknapcb2	5.250.00	59,312	250	5
	5.250.01	61,472	250	5
	5.250.02	62,130	250	5
	5.250.03	59,463	250	5
	5.250.04	58,951	250	5
mknapcb3	5.500.00	120,148	500	5
	5.500.01	117,879	500	5
	5.500.02	121,131	500	5
	5.500.03	120,804	500	5
	5.500.04	122,319	500	5
mknapcb4	10.100.00	23,064	100	10
	10.100.01	22,801	100	10
	10.100.02	22,131	100	10
	10.100.03	22,772	100	10
	10.100.04	22,751	100	10
mknapcb5	10.250.00	59,187	250	10
	10.250.01	58,781	250	10
	10.250.02	58,097	250	10
	10.250.03	61,000	250	10
	10.250.04	58,092	250	10
mknapcb6	10.500.00	117,821	500	10
	10.500.01	119,249	500	10
	10.500.02	119,215	500	10
	10.500.03	118,829	500	10
	10.500.04	116,530	500	10

**Table 3:** The second experimentation phase’s configuration parameters for LBLP, Classic SHO, and Classic SHO + IRace.

	MCDP		SCP		MKP	
**Algorithms**	**Parameters**	**Values**	**Parameters**	**Values**	**Parameters**	**Values**
Classic SHO	Search Agents	30	Search Agents	30	Search Agents	30
	Control Parameter (h)	[5, 0]	Control Parameter (h)	[5, 0]	Control Parameter (h)	[5, 0]
	M Constant	[0.5, 1]	M Constant	[0.5, 1]	M Constant	[0.5, 1]
	Number of Generations	10,000	Number of Generations	10,000	Number of Generations	10,000
Classic SHO + IRace	Search Agents	33	Search Agents	41	Search Agents	30
	Control Parameter (h)	[5, 0]	Control Parameter (h)	[5, 0]	Control Parameter (h)	[5, 0]
	M Constant	[0.5, 1]	M Constant	[0.5, 1]	M Constant	[0.5, 1]
	Number of Generations	10,000	Number of Generations	10,000	Number of Generations	10,000
LBLP	Search Agents	Schemes (20, 30, 40, 50)	Search Agents	Schemes (20, 30, 40, 50)	Search Agents	Schemes (20, 30, 40, 50)
	Control Parameter (h)	[5, 0]	Control Parameter (h)	[5, 0]	Control Parameter (h)	[5, 0]
	M Constant	[0.5, 1]	M Constant	[0.5, 1]	M Constant	[0.5, 1]
	Number of Generations	10,000	Number of Generations	10,000	Number of Generations	10,000
	α	100	α	100	α	100
	β	1000	β	1000	β	1000

**Table 4:** Computational results achieved by LBLP and state-of-the-art approaches solving the MCDP.

ID	Sopt	LBLP			BCSO			EVOA			MBFA			HBBO-AS		
		**Best**	**Mean**	**RPD (%)**	**Best**	**Mean**	**RPD (%)**	**Best**	**Mean**	**RPD (%)**	**Best**	**Mean**	**RPD (%)**	**Best**	**Mean**	**RPD (%)**
CFP01	0	**0**	**0.0**	0.00	**0**	0	0.00	**0**	0	0.00	**0**	0	0.00	**0**	0.00	0.00
CFP02	3	**3**	**3.7**	0.00	**3**	3	0.00	**3**	3	0.00	**3**	3	0.00	**3**	3.00	0.00
CFP03	5	**5**	**5.3**	0.00	**5**	5	0,00	**5**	5	0.00	**5**	5	0.00	**5**	5.00	0.00
CFP04	2	**2**	**2.2**	0.00	**2**	2	0.00	**2**	2	0.00	**2**	2	0.00	**2**	2.00	0.00
CFP05	8	**8**	**8.8**	0.00	**8**	8	0.00	**8**	8	0.00	9	9	12.50	**8**	8.00	0.00
CFP06	4	**4**	**4.8**	0.00	**4**	4	0.00	**4**	4	0.00	**4**	4	0.00	**4**	4.00	0.00
CFP07	7	**7**	**7.0**	0.00	**7**	7	0.00	**7**	7	0.00	8	8	14.29	**7**	7.00	0.00
CFP08	7	**7**	**7.1**	0.00	**7**	7	0.00	**7**	7	0.00	**7**	7	0.00	**7**	7.00	0.00
CFP09	25	**25**	**26.6**	0.00	**25**	25	0,00	**25**	25	0.00	27	27	8.00	**25**	25.00	0.00
CFP10	0	**0**	**0.0**	0.00	**0**	0	0.00	**0**	1.2	0.00	3	3	0.00	**0**	0.00	0.00
CFP11	0	**0**	**0.0**	0.00	**0**	0	0.00	**0**	0.8	0.00	**0**	0	0.00	**0**	0.00	0.00
CFP12	7	**7**	**7.9**	0.00	**7**	7	0.00	11	13.3	57.14	9	10.1	28.57	8	9.39	14.28
CFP13	8	9	9.9	11.76	**8**	8	0.00	12	14.3	50.00	**8**	8.4	0.00	9	9.60	12.50
CFP14	—	**24**	25.4	—	**24**	24	—	30	32.9	—	36	39.6	—	29	31.20	—
CFP15	—	**17**	17.0	—	**17**	17	—	31	35.7	—	18	21.1	—	**17**	22.79	—
CFP16	—	30	30.3	—	**29**	29.05	—	42	44.6	—	39	43.8	—	36	38.00	—
CFP17	—	**26**	26.8	—	**26**	26.53	—	32	34.2	—	32	33.2	—	33	34.20	—
CFP18	—	42	43.4	—	**41**	41.18	—	46	49.9	—	52	56.2	—	48	49.00	—
CFP19	—	40	40.7	—	**38**	38	—	51	53.4	—	49	51.6	—	50	52.20	—
CFP20	—	**2**	2.0	—	**2**	2	—	28	36	—	7	12.3	—	28	33.40	—
CFP21	—	37	37.8	—	**35**	35	—	57	60.3	—	43	43.5	—	56	58.79	—
CFP22	—	**0**	0.0	—	**0**	4.9	—	30	37.5	—	**0**	15.5	—	42	44.00	—
CFP23	—	**10**	11.1	—	**10**	13.53	—	39	44.2	—	13	15	—	44	48.20	—
CFP24	—	**18**	19.9	—	**18**	20.98	—	44	49.7	—	25	27.6	—	46	49.79	—
CFP25	—	**40**	41.7	—	**40**	42.6	—	60	61.6	—	49	56.1	—	61	63.20	—
CFP26	—	**59**	61.4	—	**59**	62.15	—	68	70	—	64	65.6	—	71	71.80	—
CFP27	—	64	64.6	—	**61**	64.05	—	69	70.6	—	67	68.8	—	71	71.40	—
CFP28	—	**54**	55.2	—	**54**	54	—	84	94.1	—	76	92.1	—	99	106.19	—
CFP29	—	**91**	93.5	—	**91**	96.1	—	102	112.8	—	106	109.1	—	118	122.00	—
CFP30	—	**37**	37.4	—	**37**	42.6	—	57	59.7	—	43	58.3	—	64	65.00	—
CFP31	—	**52**	53.4	—	**52**	57.9	—	70	75.3	—	54	60.4	—	79	84.19	—
CFP32	—	68	68.8	—	**66**	72.15	—	86	87.6	—	76	77.6	—	90	93.80	—
CFP33	—	**93**	93.6	—	**93**	94.93	—	136	144.8	—	116	122.6	—	155	159.00	—
CFP34	—	259	261.2	—	**256**	256	—	352	369.2	—	325	329.5	—	386	408.20	—
CFP35	—	90	91.6	—	**83**	110.58	—	181	195.6	—	114	119.2	—	225	231.39	—
X		35.14	36.00	0.90	**34.51**	36.61	0.00	50.83	54.58	8.24	42.54	45.86	4.87	55.03	57.65	2.06

**Table 5:** Computational results achieved by LBLP and state-of-the-art approaches solving the SCP.

ID	Sopt	LBLP			BCSO			BFO			BSFLA			BABC			BELA		
		**Best**	**Mean**	**RPD (%)**	**Best**	**Mean**	**RPD (%)**	**Best**	**Mean**	**RPD (%)**	**Best**	**Mean**	**RPD (%)**	**Best**	**Mean**	**RPD (%)**	**Best**	**Mean**	**RPD (%)**
4.1	429	**429**	**432**	**0.00**	459	480	7.00	**429**	430	**0.00**	430	430	0.23	430	430	0.23	447	448	4.20
4.2	512	**512**	**517**	**0.00**	570	594	11.30	517	517	0.97	516	518	0.78	513	513	0.20	559	559	9.18
4.3	516	**516**	**521**	**0.00**	590	607	14.30	519	522	0.58	520	520	0.78	519	521	0.58	537	539	4.07
4.4	494	**494**	**503**	**0.00**	547	578	10.70	495	497	0.20	501	504	1.42	495	496	0.20	527	530	6.68
4.5	512	514	517	0.39	545	554	6.40	514	515	0.39	514	514	0.39	514	517	0.39	527	529	2.93
4.6	560	**560**	**560**	**0.00**	637	650	13.80	563	565	0.53	563	563	0.54	561	565	0.18	607	608	8.39
4.7	430	**430**	**432**	**0.00**	462	467	7.40	**430**	430	**0.00**	431	432	0.23	431	434	0.23	448	449	4.19
4.8	492	**492**	**495**	**0.00**	546	567	11.00	497	499	1.01	497	499	1.02	493	494	0.20	509	512	3.46
4.9	645	**645**	**648**	**0.00**	711	725	10.90	655	658	2.18	656	656	2.34	649	651	0.93	682	682	6.40
4.10	514	517	526	0.58	537	552	4.50	519	523	0.97	518	519	0.78	517	519	0.58	571	571	11.09
5.1	253	**253**	**255**	**0.00**	279	287	10.30	257	260	1.58	254	255	0.40	254	255	0.40	280	281	10.67
5.2	302	309	309	2.29	339	340	12.30	309	311	2.31	307	307	1.66	309	309	2.32	318	321	5.30
5.3	226	**226**	**230**	**0.00**	247	251	9.30	229	233	1.32	228	230	0.88	229	233	1.33	242	240	7.08
5.4	242	**242**	**245**	**0.00**	251	253	3.70	**242**	**242**	**0.00**	**242**	**242**	**0.00**	**242**	245	**0.00**	251	252	3.72
5.5	211	**211**	**213**	**0.00**	230	230	9.00	**211**	213	**0.00**	**211**	213	**0.00**	**211**	212	**0.00**	225	227	6.64
5.6	213	**213**	**213**	**0.00**	232	243	8.90	**213**	**213**	**0.00**	**213**	214	**0.00**	214	214	0.47	247	248	15.96
5.7	293	297	301	1.36	332	338	13.30	298	301	1.70	297	299	1.37	298	301	1.71	316	317	7.85
5.8	288	**288**	**291**	**0.00**	320	330	11.10	291	292	1.04	291	293	1.04	289	291	0.35	315	317	9.38
5.9	279	280	281	0.36	295	297	5.70	284	284	1.79	281	283	0.72	280	281	0.36	314	315	12.54
5.10	265	**265**	**267**	**0.00**	285	287	7.50	268	270	1.13	**265**	266	**0.00**	267	270	0.75	280	282	5.66
6.1	138	142	144	2.86	151	160	9.40	**138**	140	**0.00**	140	141	1.45	142	143	2.90	152	152	10.14
6.2	146	**146**	**150**	**0.00**	152	157	4.10	147	149	0.68	147	147	0.68	147	150	0.68	160	161	9.59
6.3	145	**145**	**148**	**0.00**	160	164	10.30	147	150	1.37	147	148	1.38	148	149	2.07	160	163	10.34
6.4	131	**131**	**133**	**0.00**	138	142	5.30	**131**	**131**	**0.00**	**131**	133	**0.00**	**131**	133	**0.00**	140	142	6.87
6.5	161	**161**	**161**	**0.00**	169	173	5.00	164	157	1.86	166	169	3.11	165	167	2.48	184	187	14.29
A.1	253	**253**	**256**	**0.00**	286	287	13.00	255	256	0.79	255	258	0.79	254	254	0.40	261	264	3.16
A.2	252	256	257	**1.57**	274	276	8.70	259	261	2.77	260	260	3.17	257	259	1.98	279	281	10.71
A.3	232	233	235	0.43	257	263	10.80	238	240	2.58	237	239	2.16	235	238	1.29	252	253	8.62
A.4	234	235	239	0.43	248	251	6.00	235	237	0.42	235	238	0.43	236	237	0.85	250	252	6.84
A.5	236	**236**	**237**	**0.00**	244	244	3.00	**236**	237	**0.00**	**236**	239	**0.00**	**236**	238	**0.00**	241	243	2.12
B.1	69	**69**	**71**	**0.00**	79	79	14.50	71	72	2.89	70	70	1.45	70	70	1.45	86	87	24.64
B.2	76	**76**	**78**	**0.00**	86	89	13.20	78	78	2.63	**76**	77	**0.00**	78	79	2.63	88	88	15.79
B.3	80	**80**	**80**	**0.00**	85	85	6.30	**80**	**80**	**0.00**	**80**	**80**	**0.00**	**80**	**80**	**0.00**	85	87	6.25
B.4	79	**79**	**80**	**0.00**	89	89	12.70	80	81	1.26	**79**	80	**0.00**	80	81	1.27	84	88	6.33
B.5	72	**72**	**74**	**0.00**	73	73	1.40	**72**	73	**0.00**	**72**	73	**0.00**	**72**	74	**0.00**	78	81	8.33
C.1	227	229	232	0.88	242	242	6.60	230	232	1.32	229	231	0.88	231	233	1.76	237	238	4.41
C.2	219	221	225	0.91	240	241	9.60	223	224	1.82	223	225	1.83	222	223	1.37	237	239	8.22
C.3	243	**243**	**256**	**0.00**	277	278	14.00	253	254	4.11	253	253	4.12	254	255	4.53	271	271	11.52
C.4	219	**219**	**222**	**0.00**	250	250	12.30	225	227	2.73	227	228	3.65	231	233	5.48	246	248	12.33
C.5	215	**215**	**219**	**0.00**	243	244	13.00	217	219	0.93	217	218	0.93	216	217	0.47	224	225	4.19
D.1	60	**60**	**61**	**0.00**	65	66	8.30	**60**	61	**0.00**	**60**	62	**0.00**	**60**	61	**0.00**	62	62	3.33
D.2	66	**66**	**66**	**0.00**	70	70	6.10	68	68	3.03	67	68	1.52	68	68	3.03	73	74	10.61
D.3	72	73	77	1.38	79	81	9.70	75	77	4.16	75	77	4.17	76	77	5.56	79	81	9.72
D.4	62	63	64	1.60	64	67	3.20	**62**	**62**	**0.00**	63	65	1.61	63	65	1.61	67	69	8.06
D.5	61	**61**	**62**	**0.00**	65	66	6.60	63	63	3.27	63	66	3.28	63	66	3.28	66	67	8.20
E.1	29	**29**	**30**	**0.00**	**29**	30	**0.00**	**29**	31	**0.00**	**29**	**29**	**0.00**	**29**	33	**0.00**	30	31	3.45
E.2	30	32	32	6.45	34	34	13.30	32	32	6.66	31	32	3.33	32	32	6.67	35	35	16.67
E.3	27	28	29	3.64	31	32	14.80	29	30	7.40	28	28	3.70	29	31	7.41	34	34	25.93
E.4	28	29	30	3.51	32	33	14.30	29	31	3.57	29	30	3.57	29	30	3.57	33	34	17.84
E.5	28	**28**	**30**	**0.00**	30	30	7.10	29	29	3.57	**28**	31	**0.00**	29	32	3.57	30	31	7.14
F.1	14	**14**	**15**	**0.00**	17	17	21.40	15	17	7.14	15	15	7.14	**14**	15	**0.00**	17	17	21.43
F.2	15	**15**	**15**	**0.00**	18	18	20.00	16	16	6.66	**15**	**15**	**0.00**	16	16	6.67	18	18	20.00
F.3	14	16	16	13.33	17	17	21.40	16	17	14.28	16	17	14.29	16	17	14.29	17	18	21.49
F.4	14	**14**	**16**	**0.00**	17	17	21.40	15	18	7.14	15	16	7.14	15	17	7.14	17	19	21.43
F.5	13	14	15	7.41	15	16	15.40	15	19	15.38	15	17	15.38	15	16	15.38	16	17	23.08
G.1	176	**176**	**178**	**0.00**	190	193	8.00	185	191	5.11	182	183	3.41	183	184	3.98	194	196	10.23
G.2	154	158	163	2.56	165	166	7.10	161	163	4.54	161	161	4.55	162	163	5.19	176	176	14.29
G.3	166	169	170	1.79	187	188	20.60	175	177	5.42	173	174	4.22	174	175	4.82	184	185	10.84
G.4	168	170	171	1.18	179	183	6.50	176	176	4.76	173	177	2.98	175	177	4.17	196	197	16.67
G.5	168	**168**	**170**	**0.00**	181	184	7.70	177	181	5.35	174	174	3.57	179	181	6.55	198	199	17.86
H.1	63	66	67	4.65	70	71	11.10	69	70	9.52	68	69	7.94	70	71	11.11	70	71	11.11
H.2	63	65	68	3.13	67	67	6.30	66	66	4.76	66	66	4.76	69	72	9.52	71	71	12.70
H.3	59	62	65	4.96	68	70	15.30	65	67	10.16	62	63	5.08	66	67	11.86	68	70	15.25
H.4	58	59	60	1.71	66	67	13.80	63	65	6.77	63	64	8.62	64	64	10.34	70	72	20.69
H.5	55	56	61	1.80	61	62	10.90	59	60	7.27	59	61	7.27	60	61	9.09	69	69	25.45
X	196.40	**197.31**	199.75	1.09	214.98	219.42	10.12	199.51	200.92	2.95	199.15	200.37	2.43	199.32	200.85	3.04	212.42	213.69	10.82

**Table 6:** Computational results achieved by LBLP and state-of-the-art approaches solving the MKP.

Instance	Test Problem	Sopt	LBLP			QPSO			3R—PSO			F & F		
			**Best**	**Mean**	**RPD (%)**	**Best**	**Mean**	**RPD (%)**	**Best**	**Mean**	**RPD (%)**	**Best**	**Mean**	**RPD (%)**
	5.100.00	24,381	**24,381**	24,360	**0.00**	**24,381**	24381	**0.00**	**24,381**	24,381	**0.00**	**24,381**	—	**0.00**
	5.100.01	24,274	**24,274**	24,274	**0.00**	**24,274**	24,274	**0.00**	**24274**	24,274	**0.00**	**24,274**	—	**0.00**
	5.100.02	23,551	**23,551**	23,546	**0.00**	**23,551**	23,551	**0.00**	23,538	23,538	0.06	**23,551**	—	**0.00**
	5.100.03	23534	**23534**	23473	**0.00**	**23,534**	23,534	**0.00**	**23534**	23,508	**0.00**	**23,534**	—	**0.00**
mknapcb1	5.100.04	23,991	**23,991**	23,980	**0.00**	**23,991**	23,991	**0.00**	**23,991**	23,961	**0.00**	**23,991**	—	**0.00**
	5.250.00	59,312	**59,312**	58,934	**0.00**	**59,312**	59,312	**0.00**	—	—	—	**59,312**	—	**0.00**
	5.250.01	61,472	**61,472**	61,324	**0.00**	**61,472**	61,470	**0.00**	—	—	—	61,468	—	0.01
	5.250.02	62,130	**62,130**	61,997	**0.00**	**62,130**	62,130	**0.00**	—	—	—	**62,130**	—	**0.00**
	5.250.03	59,463	**59,463**	56,901	**0.00**	59,427	59,427	0.06	—	—	—	59,436	—	0.05
mknapcb2	5.250.04	58,951	58,082	57,789	1.47	**58,951**	58,951	**0.00**	—	—	—	**58,951**	—	**0.00**
	5.500.00	120,148	**120,148**	120,121	**0.00**	120,130	120,105	0.01	120,141	102,101	0.01	120,134	—	0.01
	5.500.01	117,879	115,634	114,143	1.90	117,844	117,834	0.03	117,864	117,825	0.01	117,864	—	0.01
	5.500.02	121,131	**121,131**	120,499	**0.00**	121,112	121,092	0.02	121,129	121,103	**0.00**	**121,131**	—	**0.00**
	5.500.03	120,804	119,124	117,311	1.39	**120,804**	120,740	**0.00**	**120,804**	120,722	**0.00**	120,794	—	0.01
mknapcb3	5.500.04	122,319	**122,319**	119,153	**0.00**	**122,319**	122,300	**0.00**	**122,319**	122,310	**0.00**	**122,319**	—	**0.00**
	10.100.00	23,064	**23,064**	22,981	**0.00**	**23,064**	23,064	**0.00**	**23,064**	23,050	**0.00**	**23,064**	—	**0.00**
	10.100.01	22,801	**22,801**	22,775	**0.00**	**22,801**	22,801	**0.00**	**22,801**	22,752	**0.00**	**22,801**	—	**0.00**
	10.100.02	22,131	**22,131**	22,131	**0.00**	**22,131**	22,131	**0.00**	**22,131**	22,119	**0.00**	**22,131**	—	**0.00**
	10.100.03	22,772	**22,772**	22,283	**0.00**	**22,772**	22,772	**0.00**	**22,772**	22,744	**0.00**	**22,772**	—	**0.00**
mknapcb4	10.100.04	22,751	**22,751**	22,647	**0.00**	**22,751**	22,751	**0.00**	**22,751**	22,651	**0.00**	**22,751**	—	**0.00**
	10.250.00	59,187	58,476	58,164	1.20	59,182	59173	0.01	—	—	—	59,164	—	0.04
	10.250.01	58,781	57,937	57,286	1.44	**58,781**	58,733	**0.00**	—	—	—	58,693	—	0.15
	10.250.02	58,097	**58,097**	57,921	**0.00**	**58,097**	58,096	**0.00**	—	—	—	58,094	—	0.01
	10.250.03	61000	**61,000**	60,650	**0.00**	**61,000**	60,986	**0.00**	—	—	—	60,972	—	0.05
mknapcb5	10.250.04	58,092	56,276	56,259	3.13	**58,092**	58,092	**0.00**	—	—	—	**58,092**	—	**0.00**
	10.500.00	117,821	117,779	117,754	0.04	117,744	117,733	0.07	117,790	117,699	0.03	117,734	—	0.07
	10.500.01	119,249	119,206	119,179	0.04	119,177	119,148	0.06	119,155	119,125	0.08	119,181	—	0.06
	10.500.02	119,215	**119,215**	119,162	**0.00**	**119,215**	119,146	**0.00**	119,211	119,094	**0.00**	119,194	—	0.02
	10.500.03	118,829	118,813	118,777	0.01	118,775	118,747	0.05	118,813	118,754	0.01	118,784	—	0.04
mknapcb6	10.500.04	116,530	116,509	116,470	0.02	116,502	116,449	0.02	116,470	116,509	0.05	116,471	—	0.05
X		67,455.33	67,179.10	66,741.46	0.41	**67,443.87**	67,430.47	0.02	—	—	—	67,438.93	—	0.02

**Table 7:** Computational results achieved by LBLP, Classic SHO, and Classic SHO + IRace solving the MCDP.

ID	Sopt	LBLP				Classic SHO				Classic SHO + IRace			
		**Best**	**Worst**	**Mean**	**RPD (%)**	**Best**	**Worst**	**Mean**	**RPD (%)**	**Best**	**Worst**	**Mean**	**RPD (%)**
CFP01	0	**0**	**0**	**0.0**	**0.00**	**0**	**0**	**0.0**	**0.00**	**0**	**0**	**0.0**	**0.00**
CFP02	3	**3**	5	3.7	**0.00**	**3**	4	3.5	**0.00**	**3**	4	3.6	**0.00**
CFP03	5	**5**	6	5.3	**0.00**	**5**	8	7.2	**0.00**	**5**	6	5.4	**0.00**
CFP04	2	**2**	3	2.2	**0.00**	**2**	3	2.6	**0.00**	**2**	4	2.9	**0.00**
CFP05	8	**8**	10	8.8	**0.00**	**8**	9	8.6	**0.00**	**8**	8	8.0	**0.00**
CFP06	4	**4**	6	4.8	**0.00**	**4**	7	5.7	**0.00**	**4**	6	4.7	**0.00**
CFP07	7	**7**	7	7.0	**0.00**	**7**	10	8.6	**0.00**	**7**	7	7.0	**0.00**
CFP08	7	**7**	8	7.1	**0.00**	**7**	9	8.5	**0.00**	**7**	7	7.0	**0.00**
CFP09	25	**25**	28	26.6	**0.00**	**25**	27	26.0	**0.00**	**25**	27	26.2	**0.00**
CFP10	0	**0**	**0**	**0.0**	**0.00**	**0**	**0**	**0.0**	**0.00**	**0**	**0**	**0.0**	**0.00**
CFP11	0	**0**	**0**	**0.0**	**0.00**	**0**	**0**	**0.0**	**0.00**	**0**	**0**	**0.0**	**0.00**
CFP12	7	**7**	9	7.9	**0.00**	8	9	8.7	13.33	**7**	7	7.0	**0.00**
CFP13	8	9	11	9.9	11.76	10	13	12.3	22.22	9	9	9.0	11.76
CFP14	—	**24**	27	25.4	—	25	28	27.4	—	**24**	24	24.0	—
CFP15	—	**17**	17	17.0	—	19	20	19.7	—	18	19	18.5	—
CFP16	—	30	33	30.3	—	33	35	34.4	—	31	33	32.2	—
CFP17	—	**26**	28	26.8	—	26	27	26.8	—	**26**	28	27.1	—
CFP18	—	42	45	43.4	—	43	46	44.8	—	42	44	43.2	—
CFP19	—	40	43	40.7	—	43	45	44.3	—	41	41	41.0	—
CFP20	—	**2**	2	2.0	—	2	4	3.5	—	**2**	4	2.8	—
CFP21	—	37	39	37.8	—	40	41	40.8	—	38	40	38.9	—
CFP22	—	**0**	0	0.0	—	**0**	2	0.4	—	**0**	1	0.7	—
CFP23	—	**10**	14	11.1	—	12	14	13.6	—	11	13	12.0	—
CFP24	—	**18**	22	19.9	—	21	22	21.8	—	19	20	19.4	—
CFP25	—	**40**	45	41.7	—	41	43	41.8	—	**40**	42	40.8	—
CFP26	—	**59**	65	61.4	—	**59**	59	59.0	—	**59**	61	60.2	—
CFP27	—	64	68	64.6	—	65	66	65.4	—	64	64	64.0	—
CFP28	—	**54**	57	55.2	—	**54**	57	56.1	—	**54**	56	55.0	—
CFP29	—	**91**	96	93.5	—	93	94	93.7	—	92	94	93.0	—
CFP30	—	**37**	40	37.4	—	38	40	39.1	—	**37**	38	37.5	—
CFP31	—	**52**	55	53.4	—	53	54	53.5	—	**52**	52	52.0	—
CFP32	—	68	71	68.8	—	69	72	70.6	—	68	69	68.6	—
CFP33	—	**93**	95	93.6	—	95	95	95.0	—	94	96	95.0	—
CFP34	—	259	263	261.2	—	259	260	259.6	—	259	259	259.0	—
CFP35	—	90	96	91.6	—	92	94	93.6	—	91	91	91.0	—
X	5.85	**35.14**	37.54	36.00	0.90	36.09	37.57	37.04	2.74	35.43	36.37	35.90	0.90

**Table 8:** Computational results achieved by LBLP and HBBO + AS solving the MCDP.

ID	Sopt	LBLP				HBBO + AS			
		**Best**	**Worst**	**Mean**	**RPD (%)**	**Best**	**Worst**	**Mean**	**RPD (%)**
CFP01	0	**0**	**0**	**0.0**	**0.00**	**0**	0	0.0	**0.00**
CFP02	3	**3**	5	3.7	**0.00**	**3**	3	3.0	**0.00**
CFP03	5	**5**	6	5.3	**0.00**	**5**	5	5.0	**0.00**
CFP04	2	**2**	3	2.2	**0.00**	**2**	2	2.0	**0.00**
CFP05	8	**8**	10	8.8	**0.00**	**8**	8	8.0	**0.00**
CFP06	4	**4**	6	4.8	**0.00**	**4**	4	4.0	**0.00**
CFP07	7	**7**	7	7.0	**0.00**	**7**	7	7.0	**0.00**
CFP08	7	**7**	8	7.1	**0.00**	**7**	7	7.0	**0.00**
CFP09	25	**25**	28	26.6	**0.00**	**25**	25	25.0	**0.00**
CFP10	0	**0**	**0**	**0.0**	**0.00**	**0**	0	0.0	**0.00**
CFP11	0	**0**	**0**	**0.0**	**0.00**	**0**	0	0.0	**0.00**
CFP12	7	**7**	9	7.9	**0.00**	8	11	9.4	14.28
CFP13	8	9	11	9.9	11.76	9	11	9.6	12.5
CFP14	—	**24**	27	25.4	—	29	33	31.2	—
CFP15	—	**17**	17	17.0	—	17	26	22.8	—
CFP16	—	30	33	30.3	—	36	42	38.0	—
CFP17	—	**26**	28	26.8	—	33	35	34.2	—
CFP18	—	42	45	43.4	—	48	50	49.0	—
CFP19	—	40	43	40.7	—	50	54	52.2	—
CFP20	—	**2**	2	2.0	—	28	39	33.4	—
CFP21	—	37	39	37.8	—	56	63	58.8	—
CFP22	—	**0**	0	0.0	—	42	47	44.0	—
CFP23	—	**10**	14	11.1	—	44	53	48.2	—
CFP24	—	**18**	22	19.9	—	46	52	49.8	—
CFP25	—	**40**	45	41.7	—	61	67	63.2	—
CFP26	—	**59**	65	61.4	—	71	73	71.8	—
CFP27	—	64	68	64.6	—	71	72	71.4	—
CFP28	—	**54**	57	55.2	—	99	112	106.2	—
CFP29	—	**91**	96	93.5	—	118	125	122.0	—
CFP30	—	**37**	40	37.4	—	64	67	65.0	—
CFP31	—	**52**	55	53.4	—	79	89	84.2	—
CFP32	—	68	71	68.8	—	90	97	93.8	—
CFP33	—	**93**	95	93.6	—	155	167	159.0	—
CFP34	—	259	263	261.2	—	386	417	408.2	—
CFP35	—	90	96	91.6	—	225	234	231.4	—
X	5.85	**35.14**	37.54	36.00	0.90	55.03	59.91	57.65	2.06

**Table 9:** Average Wilcoxon–Mann–Whitney test for MCDP.

Approach	LBLP	Classic SHO	Classic SHO + IRace
LBLP	-	**0.00**	≥0.05
Classic SHO	≥0.05	-	≥0.05
Classic SHO + IRace	≥0.05	**0.00**	-

**Table 10:** Computational results achieved by LBLP, Classic SHO, and Classic SHO + IRace solving the SCP.

ID	Sopt	LBLP			Classic SHO			Classic SHO + IRace		
		**Best**	**Mean**	**RPD (%)**	**Best**	**Mean**	**RPD (%)**	**Best**	**Mean**	**RPD (%)**
4.1	429	**429**	432	**0.00**	430	432	**0.23**	**429**	431	**0.00**
4.2	512	**512**	517	**0.00**	528	528	**3.08**	**512**	513	**0.00**
4.3	516	**516**	521	**0.00**	532	532	**3.05**	518	518	0.39
4.4	494	**494**	503	**0.00**	505	506	**2.20**	498	500	0.81
4.5	512	514	517	0.39	514	514	**0.39**	514	516	0.39
4.6	560	**560**	**560**	**0.00**	**560**	562	**0.00**	**560**	561	**0.00**
4.7	430	**430**	432	**0.00**	**430**	430	**0.00**	**430**	432	**0.00**
4.8	492	**492**	495	**0.00**	503	503	**2.21**	**492**	493	**0.00**
4.9	645	**645**	648	**0.00**	669	669	**3.65**	655	657	1.54
4.10	514	517	526	0.58	518	518	**0.78**	517	519	0.58
5.1	253	**253**	255	**0.00**	257	257	**1.57**	**253**	255	**0.00**
5.2	302	309	309	2.29	312	314	**3.26**	310	312	2.61
5.3	226	**226**	230	**0.00**	234	234	**3.48**	**226**	**226**	**0.00**
5.4	242	**242**	245	**0.00**	**242**	242	**0.00**	**242**	244	**0.00**
5.5	211	**211**	213	**0.00**	**211**	211	**0.00**	**211**	**211**	**0.00**
5.6	213	**213**	**213**	**0.00**	216	217	**1.40**	214	216	0.47
5.7	293	297	301	1.36	296	296	**1.02**	297	299	1.36
5.8	288	**288**	291	**0.00**	291	292	**1.04**	289	291	0.35
5.9	279	280	281	0.36	280	281	**0.36**	280	282	0.36
5.10	265	**265**	267	**0.00**	271	271	**2.24**	267	268	0.75
6.1	138	142	144	2.86	140	140	**1.44**	141	143	2.15
6.2	146	**146**	150	**0.00**	**146**	146	**0.00**	**146**	**146**	**0.00**
6.3	145	**145**	148	**0.00**	148	148	**2.05**	146	147	0.69
6.4	131	**131**	133	**0.00**	133	133	**1.52**	132	133	0.76
6.5	161	**161**	**161**	**0.00**	165	166	**2.45**	163	163	1.23
A.1	253	**253**	256	**0.00**	256	256	**1.18**	254	256	0.39
A.2	252	256	257	**1.57**	259	259	**2.74**	257	258	1.96
A.3	232	233	235	0.43	239	239	**2.97**	235	235	1.28
A.4	234	235	239	0.43	235	235	**0.43**	235	236	0.43
A.5	236	**236**	237	**0.00**	**236**	236	**0.00**	**236**	237	**0.00**
B.1	69	**69**	71	**0.00**	72	72	**4.26**	70	72	1.44
B.2	76	**76**	78	**0.00**	81	81	**6.37**	78	78	2.60
B.3	80	**80**	**80**	**0.00**	**80**	80	**0.00**	**80**	82	**0.00**
B.4	79	**79**	80	**0.00**	81	82	**2.50**	80	81	1.26
B.5	72	**72**	74	**0.00**	**72**	72	**0.00**	**72**	73	**0.00**
C.1	227	229	232	0.88	233	234	**2.61**	231	233	1.75
C.2	219	221	225	0.91	223	223	**1.81**	222	222	1.36
C.3	243	**243**	256	**0.00**	251	251	**3.24**	246	246	1.23
C.4	219	**219**	222	**0.00**	225	225	**2.70**	221	221	0.91
C.5	215	**215**	219	**0.00**	**215**	215	**0.00**	**215**	217	**0.00**
D.1	60	**60**	61	**0.00**	**60**	60	**0.00**	**60**	**60**	**0.00**
D.2	66	**66**	**66**	**0.00**	68	68	**2.99**	67	67	1.50
D.3	72	73	77	1.38	76	76	**5.41**	74	76	2.74
D.4	62	63	64	1.60	**62**	62	**0.00**	63	64	1.60
D.5	61	**61**	62	**0.00**	**61**	61	**0.00**	**61**	63	**0.00**
E.1	29	**29**	30	**0.00**	**29**	29	**0.00**	**29**	29	**0.00**
E.2	30	32	32	6.45	31	31	**3.28**	32	33	6.45
E.3	27	28	29	3.64	27	27	**0.00**	28	28	3.64
E.4	28	29	30	3.51	29	29	**3.51**	29	30	3.51
E.5	28	**28**	30	**0.00**	**28**	28	**0.00**	**28**	30	**0.00**
F.1	14	**14**	15	**0.00**	**14**	14	**0.00**	**14**	**14**	**0.00**
F.2	15	**15**	**15**	**0.00**	**15**	15	**0.00**	**15**	**15**	**0.00**
F.3	14	16	16	13.33	17	18	**19.35**	16	18	13.33
F.4	14	**14**	16	**0.00**	15	16	**6.90**	**14**	**14**	**0.00**
F.5	13	14	15	7.41	14	16	**7.41**	14	16	7.41
G.1	176	**176**	178	**0.00**	178	180	**1.13**	177	178	0.57
G.2	154	158	163	2.56	158	159	**2.56**	158	160	2.56
G.3	166	169	170	1.79	170	172	**2.38**	169	170	1.79
G.4	168	170	171	1.18	**168**	168	**0.00**	**168**	**168**	**0.00**
G.5	168	**168**	170	**0.00**	170	170	**1.18**	**168**	169	**0.00**
H.1	63	66	67	4.65	68	70	**7.63**	66	66	4.65
H.2	63	65	68	3.13	65	66	**3.13**	65	67	3.13
H.3	59	62	65	4.96	62	65	**4.96**	62	63	4.96
H.4	58	59	60	1.71	**58**	58	**0.00**	59	59	1.71
H.5	55	56	61	1.80	58	60	**5.31**	57	58	3.57
X	196.40	**197.31**	199.75	1.09	199.85	200.31	2.24	197.95	199.05	1.42

**Table 11:** Average Wilcoxon-Mann-Whitney test for SCP.

Approach	LBLP	Classic SHO	Classic SHO + IRace
LBLP	-	**0.00**	≥0.05
Classic SHO	≥0.05	-	≥0.05
Classic SHO + IRace	≥0.05	**0.00**	-

**Table 12:** Computational results achieved by LBLP, Classic SHO, and Classic SHO + IRace solving the MKP.

ID	Test Problem	Sopt	LBLP				Classic SHO				Classic SHO + IRace			
			**Best**	**Worst**	**Mean**	**RPD (%)**	**Best**	**Worst**	**Mean**	**RPD (%)**	**Best**	**Worst**	**Mean**	**RPD (%)**
mknapcb1	5.100.00	24,381	**24,381**	24,301	24,360	**0.00**	**24,381**	22,431	23,796	**0.00**	**24,381**	22,674	24,279	**0.00**
	5.100.01	24,274	**24,274**	**24,274**	**24,274**	**0.00**	**24,274**	24,274	24,274	**0.00**	**24274**	24,274	24,274	**0.00**
	5.100.02	23,551	**23,551**	23,538	23,546	**0.00**	**23,551**	21,196	22,868	**0.00**	**23,551**	22,138	23,311	**0.00**
	5.100.03	23,534	**23,534**	23,288	23,473	**0.00**	**23,534**	21,181	22,828	**0.00**	**23,534**	21,887	23,188	**0.00**
	5.100.04	23,991	**23,991**	23,947	23,980	**0.00**	**23,991**	21,832	23,624	**0.00**	**23,991**	23,031	23,809	**0.00**
mknapcb2	5.250.00	59,312	**59,312**	58,473	58,934	**0.00**	**59,312**	55,160	58,066	**0.00**	**59,312**	55,753	58,814	**0.00**
	5.250.01	61,472	**61,472**	60,692	61,324	**0.00**	**61,472**	55,325	60,857	**0.00**	**61,472**	59,628	61,361	**0.00**
	5.250.02	62,130	**62,130**	61,702	**61,997**	**0.00**	60,266	56,650	59,398	3.00	**62,130**	57,781	61,695	**0.00**
	5.250.03	59,463	**59,463**	55,164	56,901	**0.00**	**59,463**	53,517	57,739	**0.00**	**59,463**	57,084	59,082	**0.00**
	5.250.04	58,951	58,082	57,550	57,789	1.47	56,003	53,203	55,611	5.00	58,361	56,611	58,204	1.00
mknapcb3	5.500.00	120,148	**120,148**	119,978	120,121	**0.00**	**120,148**	108,133	118,826	**0.00**	119,908	113,912	118,769	0.20
	5.500.01	117,879	115,634	112,821	114,143	1.90	**117,879**	109,627	116,971	**0.00**	**117,879**	111,985	116,523	**0.00**
	5.500.02	121,131	**121,131**	119,156	120,499	**0.00**	120,525	114,499	118,778	0.50	**121,131**	112,652	120,368	**0.00**
	5.500.03	120,804	119,124	115,828	117,311	1.39	120,200	114,190	119,479	0.50	**120,804**	114,764	119,415	**0.00**
	5.500.04	122,319	**122,319**	117,242	119,153	**0.00**	121,707	111,971	119,079	0.50	122,074	113,529	120024	0.20
mknapcb4	10.100.00	23,064	**23,064**	22,905	22,981	**0.00**	21,911	20,815	21,659	5.00	**23,064**	22,372	22,974	**0.00**
	10.100.01	22,801	**22,801**	22,630	22,775	**0.00**	**22,801**	21,205	22,434	**0.00**	**22,801**	21,205	22,625	**0.00**
	10.100.02	22,131	**22,131**	**22,131**	**22,131**	**0.00**	**22,131**	21,024	21,976	**0.00**	**22,131**	20,803	22,065	**0.00**
	10.100.03	22,772	**22,772**	22,052	22,283	**0.00**	**22,772**	21,633	22,556	**0.00**	**22,772**	21,178	22,629	**0.00**
	10.100.04	22,751	**22,751**	22,417	22,647	**0.00**	**22,751**	21,158	22,273	**0.00**	**22,751**	21,613	22,535	**0.00**
mknapcb5	10.250.00	59,187	58,476	57,530	58,164	1.20	56,820	51,138	55,569	4.00	58,003	56,263	57,777	2.00
	10.250.01	58,781	57,937	56,490	57,286	1.44	55,842	51,933	54,865	5.00	**58,781**	56,430	58,287	**0.00**
	10.250.02	58,097	**58,097**	57,062	57,921	**0.00**	55,773	51,311	54,435	4.00	**58,097**	54,611	57,226	**0.00**
	10.250.03	61,000	**61,000**	60,326	60,650	**0.00**	**61,000**	56,730	60,402	**0.00**	59,170	54,436	58,271	3.00
	10.250.04	58,092	56,276	56,204	56,259	3.13	55,768	50,749	55,066	4.00	57,511	54,636	56,965	1.00
mknapcb6	10.500.00	117,821	117,779	117,736	117,754	0.04	117,232	110,198	116,317	0.50	117,585	111,706	116,703	0.20
	10.500.01	119,249	119,206	119,164	119,179	0.04	118,534	107,865	115,867	0.60	**119,249**	109,709	117,627	0.00
	10.500.02	119,215	**119,215**	119,129	119,162	**0.00**	118,619	110,316	116,294	0.50	118,857	114,103	118,620	0.30
	10.500.03	118,829	118,813	118,761	118,777	0.01	117,878	109,627	116,558	0.80	118,710	112,775	117,345	0.10
	10.500.04	116,530	116,509	116,445	116,470	0.02	115,365	106,136	112,781	1.00	116,297	108,156	115,157	0.20
X		67,455.33	67,179	66,298	66,741	0.35	66,730	61,834	65,708	1.16	**67,268**	63,590	66,664	0.27

**Table 13:** Computational results achieved by LBLP, and LMPB solving the MKP.

ID	Test Problem	Sopt	LBLP				LMPB			
			**Best**	**Worst**	**Mean**	**RPD (%)**	**Best**	**Worst**	**Mean**	**RPD (%)**
mknapcb1	5.100.00	24,381	**24,381**	24,301	24,360	**0.00**	**24,381**	17,595	18,193	**0.00**
	5.100.01	24,274	**24,274**	**24,274**	**24,274**	**0.00**	**24,274**	17,401	17,674	**0.00**
	5.100.02	23,551	**23,551**	23,538	23,546	**0.00**	**23,551**	17,692	17,861	**0.00**
	5.100.03	23,534	**23,534**	23,288	23,473	**0.00**	**23,534**	19,685	19,692	**0.00**
	5.100.04	23,991	**23,991**	23,947	23,980	**0.00**	**23,991**	17,744	17,863	**0.00**
mknapcb2	5.250.00	59,312	**59,312**	58,473	58,934	**0.00**	**59,312**	46,049	46,588	**0.00**
	5.250.01	61,472	**61,472**	60,692	61,324	**0.00**	**61,472**	46,890	47,299	**0.00**
	5.250.02	62,130	**62,130**	61,702	**61,997**	**0.00**	**62,130**	49,237	49,262	**0.00**
	5.250.03	59,463	**59,463**	55,164	56,901	**0.00**	**59,463**	42,804	46,365	**0.00**
	5.250.04	58,951	58,082	57,550	57,789	1.47	**58,951**	46,870	47,005	**0.00**
mknapcb3	5.500.00	120,148	**120,148**	119,978	120,121	**0.00**	101,980	73,168	88,110	15.12
	5.500.01	117,879	115,634	112,821	114,143	1.90	99,901	71,265	90,507	15.25
	5.500.02	121,131	**121,131**	119,156	120,499	**0.00**	102,559	74,678	91,014	15.33
	5.500.03	120,804	119,124	115,828	117,311	1.39	100,864	74,715	91,769	16.5
	5.500.04	122,319	**122,319**	117,242	119,153	**0.00**	102,520	74,537	91,772	16.18
mknapcb4	10.100.00	23,064	**23,064**	22,905	22,981	**0.00**	**23,064**	17,298	22,276	**0.00**
	10.100.01	22,801	**22,801**	22,630	22,775	**0.00**	**22,801**	17,352	21,296	**0.00**
	10.100.02	22,131	**22,131**	**22,131**	**22,131**	**0.00**	**22,131**	15,699	20,487	**0.00**
	10.100.03	22,772	**22,772**	22,052	22,283	**0.00**	**22,772**	18,817	19,796	**0.00**
	10.100.04	22,751	**22,751**	22,417	22,647	**0.00**	**22,751**	17,564	22,604	**0.00**
mknapcb5	10.250.00	59,187	58,476	57,530	58,164	1.20	**59,187**	48,086	55,819	**0.00**
	10.250.01	58,781	57,937	56,490	57,286	1.44	**58,781**	43,173	55,303	**0.00**
	10.250.02	58,097	**58,097**	57062	57,921	**0.00**	**58,097**	45,538	52,908	**0.00**
	10.250.03	61,000	**61,000**	60,326	60,650	**0.00**	**61,000**	47,587	57,342	**0.00**
	10.250.04	58,092	56,276	56,204	56,259	3.13	**58,092**	47,703	55,037	**0.00**
mknapcb6	10.500.00	117,821	117,779	117,736	117,754	0.04	103,226	74,746	93,309	12.38
	10.500.01	119,249	119,206	119,164	119,179	0.04	105,088	76,531	96,824	11.87
	10.500.02	119,215	**119,215**	119,129	119,162	**0.00**	104,870	74,620	96,152	12.03
	10.500.03	118,829	118,813	118,761	118,777	0.01	104,308	74,845	95,339	12.22
	10.500.04	116,530	116,509	116,445	116,470	0.02	101,380	74,441	92,260	13
X		67,455.33	67,179	66,298	66,741	0.35	61,881.03333	46,144.33333	54,591	4.66

**Table 14:** Average Wilcoxon-Mann-Whitney test for MKP.

Approach	LBLP	Classic SHO	Classic SHO + IRace
LBLP	-	**0.00**	≥0.05
Classic SHO	≥0.05	-	≥0.05
Classic SHO + IRace	≥0.05	**0.00**	-

## Data Availability

No new data were created or analyzed in this study. Data sharing is not applicable to this article.

## References

[1] Talbi E.G. (2009). Metaheuristics: From Design to Implementation.

[2] Del Ser J., Osaba E., Molina D., Yang X.S., Salcedo-Sanz S., Camacho D., Das S., Suganthan P.N., Coello C.A., Herrera F. (2019). Bio-inspired computation: Where we stand and what’s next. Swarm Evol. Comput..

[3] Piotrowski A.P., Napiorkowski J.J., Piotrowska A.E. (2020). Population size in particle swarm optimization. Swarm Evol. Comput..

[4] Hansen N., Müller S.D., Koumoutsakos P. (2003). Reducing the time complexity of the derandomized evolution strategy with covariance matrix adaptation (CMA-ES). Evol. Comput..

[5] Hansen N., Auger A. CMA-ES: Evolution strategies and covariance matrix adaptation. Proceedings of the 13th Annual Conference Companion on Genetic and Evolutionary Computation.

[6] Sarker R., Kamruzzaman J., Newton C. (2003). Evolutionary optimization (EvOpt): A brief review and analysis. Int. J. Comput. Intell. Appl..

[7] Hansen N., Ostermeier A. (2001). Completely derandomized self-adaptation in evolution strategies. Evol. Comput..

[8] Gupta S. (2022). Enhanced harmony search algorithm with non-linear control parameters for global optimization and engineering design problems. Eng. Comput..

[9] Huang Y.F., Chen P.H. (2020). Fake news detection using an ensemble learning model based on self-adaptive harmony search algorithms. Expert Syst. Appl..

[10] Kulluk S., Ozbakir L., Baykasoglu A. (2011). Self-adaptive global best harmony search algorithm for training neural networks. Procedia Comput. Sci..

[11] Banks A., Vincent J., Anyakoha C. (2007). A review of particle swarm optimization. Part I: Background and development. Nat. Comput..

[12] Cheng S., Lu H., Lei X., Shi Y. (2018). A quarter century of particle swarm optimization. Complex Intell. Syst..

[13] Wang D., Tan D., Liu L. (2018). Particle swarm optimization algorithm: An overview. Soft Comput..

[14] Song H., Triguero I., Özcan E. (2019). A review on the self and dual interactions between machine learning and optimisation. Prog. Artif. Intell..

[15] Karimi-Mamaghan M., Mohammadi M., Meyer P., Karimi-Mamaghan A.M., Talbi E.G. (2022). Machine learning at the service of meta-heuristics for solving combinatorial optimization problems: A state-of-the-art. Eur. J. Oper. Res..

[16] Talbi E.G. (2021). Machine learning into metaheuristics: A survey and taxonomy. Acm Comput. Surv. (CSUR).

[17] Birattari M., Kacprzyk J. (2009). Tuning Metaheuristics: A Machine Learning Perspective (Vol. 197).

[18] Talbi E.G. (2016). Combining metaheuristics with mathematical programming, constraint programming and machine learning. Ann. Oper. Res..

[19] Calvet L., de Armas J., Masip D., Juan A.A. (2017). Learnheuristics: Hybridizing metaheuristics with machine learning for optimization with dynamic inputs. Open Math..

[20] Dhiman G., Kumar V. (2017). Spotted hyena optimizer: A novel bio-inspired based metaheuristic technique for engineering applications. Adv. Eng. Software.

[21] Luo Q., Li J., Zhou Y., Liao L. (2021). Using spotted hyena optimizer for training feedforward neural networks. Cogn. Syst. Res..

[22] Soto R., Crawford B., Vega E., Gómez A., Gómez-Pulido J.A., Wotawa F., Friedrich G., Pill I., Koitz-Hristov R., Ali M. (2019). Solving the Set Covering Problem Using Spotted Hyena Optimizer and Autonomous Search. Advances and Trends in Artificial Intelligence.

[23] Ghafori S., Gharehchopogh F.S. (2022). Advances in spotted hyena optimizer: A comprehensive survey. Arch. Comput. Methods Eng..

[24] Dhiman G., Kaur A. Spotted hyena optimizer for solving engineering design problems. Proceedings of the 2017 International Conference on Machine Learning and Data Science (MLDS).

[25] Dhiman G., Kumar V. (2019). Spotted hyena optimizer for solving complex and non-linear constrained engineering problems. Harmony Search and Nature Inspired Optimization Algorithms: Theory and Applications, ICHSA 2018.

[26] Dhiman G., Kaur A. (2019). A hybrid algorithm based on particle swarm and spotted hyena optimizer for global optimization. Soft Computing for Problem Solving: SocProS 2017.

[27] Mahdavi I., Paydar M.M., Solimanpur M., Heidarzade A. (2009). Genetic algorithm approach for solving a cell formation problem in cellular manufacturing. Expert Syst. Appl..

[28] Beasley J.E. (1987). An algorithm for set covering problem. Eur. J. Oper. Res..

[29] Fréville A. (2004). The multidimensional 0–1 knapsack problem: An overview. Eur. J. Oper. Res..

[30] Lopez-Ibanez M., Dubois-Lacoste J., Caceres L.P., Birattari M., Stutzle T. (2016). The irace package: Iterated racing for automatic algorithm configuration. Oper. Res. Perspect..

[31] Talbi E.-G. (2002). A taxonomy of hybrid metaheuristics. J. Heuristics.

[32] Zennaki M., Ech-Cherif A. (2010). A new machine learning based approach for tuning metaheuristics for the solution of hard combinatorial optimization problems. J. Appl. Sci..

[33] de Lacerda M.G.P., de Araujo Pessoa L.F., de Lima Neto F.B., Ludermir T.B., Kuchen H. (2021). A systematic literature review on general parameter control for evolutionary and swarm-based algorithms. Swarm Evol. Comput..

[34] Soto R., Crawford B., González F., Vega E., Castro C., Paredes F. (2019). Solving the Manufacturing Cell Design Problem Using Human Behavior-Based Algorithm Supported by Autonomous Search. IEEE Access.

[35] Handa H., Baba M., Horiuchi T., Katai O. (2002). A novel hybrid framework of coevolutionary GA and machine learning. Int. J. Comput. Intell. Appl..

[36] Adak Z., Demiriz A. (2020). Hybridization of population-based ant colony optimization via data mining. Intell. Data Anal..

[37] Streichert F., Stein G., Ulmer H., Zell A. (2003). A clustering based niching method for evolutionary algorithms. Genetic and Evolutionary Computation Conference.

[38] Sörensen K., Glover F. (2013). Metaheuristics. Encycl. Oper. Res. Manag. Sci..

[39] Gogna A., Tayal A. (2013). Metaheuristics: Review and application. J. Exp. Theor. Artif. Intell..

[40] Crama Y., Kolen A.W., Pesch E.J. (1995). Local search in combinatorial optimization. Artif. Neural Networks.

[41] Kirkpatrick S. (1984). Optimization by simulated annealing: Quantitative studies. J. Stat. Physics.

[42] Eusuff M., Lansey K., Pasha F. (2006). Shuffled frog-leaping algorithm: A memetic meta-heuristic for discrete optimization. Eng. Optim..

[43] Dorigo M., Birattari M., Stutzle T. (2006). Ant colony optimization. IEEE Comput. Intell. Mag..

[44] Mirjalili S., Mirjalili S.M., Lewis A. (2014). Grey wolf optimizer. Adv. Eng. Software.

[45] Holland J.H. (1992). Adaptation in Natural and Artificial Systems: An Introductory Analysis with Applications to Biology, Control, and Artificial Intelligence.

[46] Moscato P. (1989). On evolution, search, optimization, genetic algorithms and martial arts: Towards memetic algorithms. Caltech Concurr. Comput. Program C3p Rep..

[47] Storn R., Price K. (1997). Differential evolution—A simple and efficient heuristic for global optimization over continuous spaces. J. Glob. Optim..

[48] Atashpaz-Gargari E., Lucas C. Imperialist competitive algorithm: An algorithm for optimization inspired by imperialistic competition. Proceedings of the 2007 IEEE Congress on Evolutionary Computation.

[49] Cuevas E., Fausto F., González A. (2020). New Advancements in Swarm Algorithms: Operators and Applications.

[50] Mirjalili S., Mirjalili S.M., Yang X.S. (2014). Binary bat algorithm. Neural Comput. Appl..

[51] Mirjalili S., Lewis A. (2013). S-shaped versus v-shaped transfer functions for binary particle swarm optimization. Swarm Evol. Comput..

[52] Faris H., Mafarja M.M., Heidari A.A., Aljarah I., Ala’M A.Z., Mirjalili S., Fujita H. (2018). An efficient binary salp swarm algorithm with crossover scheme for feature selection problems. Knowl.-Based Syst..

[53] Mafarja M., Aljarah I., Heidari A.A., Faris H., Fournier-Viger P., Li X., Mirjalili S. (2018). Binary dragonfly optimization for feature selection using time-varying transfer functions. Knowl.-Based Syst..

[54] Mirjalili S., Hashim S.Z.M. (2012). BMOA: Binary magnetic optimization algorithm. Int. J. Mach. Learn. Comput..

[55] Valenzuela M., Pinto H., Moraga P., Altimiras F., Villavicencio G. (2019). A percentile methodology applied to Binarization of swarm intelligence metaheuristics. J. Inf. Syst. Eng. Manag..

[56] Gölcük İ., Ozsoydan F.B., Durmaz E.D. Analysis of Different Binarization Techniques within Whale Optimization Algorithm. Proceedings of the 2019 Innovations in Intelligent Systems and Applications Conference (ASYU).

[57] Slezkin A.O., Hodashinsky I.A., Shelupanov A.A. (2021). Binarization of the Swallow swarm optimization for feature selection. Program. Comput. Softw..

[58] Kennedy J., Eberhart R.C. A discrete binary version of the particle swarm algorithm. Proceedings of the 1997 IEEE International Conference on Systems, Man, and Cybernetics. Computational Cybernetics and Simulation.

[59] Boctor F.F. (1991). A Jinear formulation of the machine-part cell formation problem. Int. J. Prod. Res..

[60] Smith B. (1988). Impacs—A bus crew scheduling system using integer programming. Math Program.

[61] Toregas C., Swain R., Revelle C., Bergman L. (1971). The location of emergency service facilities. Oper. Res..

[62] Foster B., Ryan D. (1976). An integer programming approach to the vehicle scheduling problem. Oper. Res. Q.

[63] Fisher M., Kedia P. (1990). Optimal solution of set covering/partitioning problems using dual heuristics. Manag. Sci..

[64] Pisinger D. (2007). The quadratic knapsack problem—A survey. Discret. Appl. Math..

[65] Horowitz E., Sahni S. (1974). Computing partitions with applications to the knapsack problem. J. ACM (JACM).

[66] Soto R., Crawford B., Toledo A., Fuente-Mella H., Castro C., Paredes F., Olivares R. (2019). Solving the Manufacturing Cell Design Problem through Binary Cat Swarm Optimization with Dynamic Mixture Ratios. Comput. Intell. Neurosci..

[67] Almonacid B., Aspee F., Soto R., Crawford B., Lama J. (2016). Solving the Manufacturing Cell Design Problem using the Modified Binary Firefly Algorithm and the Egyptian Vulture Optimization Algorithm. IET Softw..

[68] Crawford B., Soto R., Berros N., Johnson F., Paredes F., Castro C., Norero E. (2014). A binary cat swarm optimization algorithm for the non-unicost set covering problem. Math Probl. Eng..

[69] Crawford B., Soto R., Olivares-Suarez M., Paredes F. (2014). A binary firefly algorithm for the set covering problem. 3rd Computer Science On-Line Conference 2014 (CSOC 2014).

[70] Crawford B., Soto R., Pena C., Palma W., Johnson F., Paredes F. (2015). Solving the set covering problem with a shuffled frog leaping algorithm. Proceedings of the 7th Asian Conference, ACIIDS 2015.

[71] Cuesta R., Crawford B., Soto R., Paredes F. (2014). An artificial bee colony algorithm for the set covering problem, In 3rd Computer Science On-Line Conference 2014 (CSOC 2014).

[72] Soto R., Crawford B., Munoz A., Johnson F., Paredes F. (2015). Preprocessing, repairing and transfer functions can help binary electromagnetism-like algorithms. Artificial Intelligence Perspectives and Applications.

[73] Khemakhem M., Haddar B., Chebil K., Hanafi S. (2012). A Filter-and-Fan Metaheuristic for the 0-1 Multidimensional Knapsack Problem. Int. J. Appl. Metaheuristic Comput. (IJAMC).

[74] Chih M. (2018). Three pseudo-utility ratio-inspired particle swarm optimization with local search for multidimensional knapsack problem. Swarm Evol. Comput..

[75] Haddar B., Khemakhem M., Hanafi S., Wilbaut C. (2016). A hybrid quantum particle swarm optimization for the multidimensional knapsack problem. Eng. Appl. Artif. Intell..

[76] Vega E., Soto R., Contreras P., Crawford B., Peña J., Castro C. (2022). Combining a Population-Based Approach with Multiple Linear Models for Continuous and Discrete Optimization Problems. Mathematics.

